# Ethylene-independent signaling by the ethylene precursor ACC in *Arabidopsis* ovular pollen tube attraction

**DOI:** 10.1038/s41467-020-17819-9

**Published:** 2020-08-14

**Authors:** Wangshu Mou, Yun-Ting Kao, Erwan Michard, Alexander A. Simon, Dongdong Li, Michael M. Wudick, Michael A. Lizzio, José A. Feijó, Caren Chang

**Affiliations:** 1 Department of Cell Biology and Molecular Genetics, University of Maryland, College Park, MD USA; 2 Present Address: Division of Crop Biotechnics, Department of Biosystems, University of Leuven, Leuven, Belgium; 3 Present Address: Institute for Molecular Physiology, Heinrich-Heine-Universität Düsseldorf, Düsseldorf, Germany

**Keywords:** Plant hormones, Plant signalling

## Abstract

The phytohormone ethylene has numerous effects on plant growth and development. Its immediate precursor, 1-aminocyclopropane-1-carboxylic acid (ACC), is a non-proteinogenic amino acid produced by ACC SYNTHASE (ACS). ACC is often used to induce ethylene responses. Here, we demonstrate that ACC exhibits ethylene-independent signaling in *Arabidopsis thaliana* reproduction. By analyzing an *acs* octuple mutant with reduced seed set, we find that ACC signaling in ovular sporophytic tissue is involved in pollen tube attraction, and promotes secretion of the pollen tube chemoattractant LURE1.2. ACC activates Ca^2+^-containing ion currents via GLUTAMATE RECEPTOR-LIKE (GLR) channels in root protoplasts. In COS-7 cells expressing moss *Pp*GLR1, ACC induces the highest cytosolic Ca^2+^ elevation compared to all twenty proteinogenic amino acids. In ovules, ACC stimulates transient Ca^2+^ elevation, and Ca^2+^ influx in octuple mutant ovules rescues LURE1.2 secretion. These findings uncover a novel ACC function and provide insights for unraveling new physiological implications of ACC in plants.

## Abstract (web summary)

Ethylene is synthesized from the non-proteinogenic amino acid ACC. Here, Mou et al. show that ACC itself acts independently of ethylene to trigger secretion of a pollen tube attractant in the sporophytic tissue of Arabidopsis ovules and can activate Ca^2+^-currents via GLUTAMATE RECEPTOR-LIKE channels.

## Introduction

Ethylene is a plant hormone that induces numerous physiological responses in the growth and development of flowering plants^1^. Ethylene biosynthesis in flowering plants starts with the synthesis of 1-aminocyclopropane-1-carboxylic acid (ACC) (a non-proteinogenic amino acid) from S-adenosylmethionine via the enzyme ACC SYNTHASE (ACS), followed by conversion of ACC to ethylene by the enzyme ACC OXIDASE (ACO)^2^. Responses to ethylene are often assessed by treating plants with ACC, due to the relative ease of application and rapid conversion of ACC to ethylene^2^. However, exceptions to this rule, such as ethylene-independent ACC responses in roots^3–5^, guard cells^6^, and reproduction^7^ have been reported. In the latter case, an *Arabidopsis thaliana* octuple mutant of all eight functional *ACS* genes was generated by expressing an artificial microRNA (amiRNA) that reduced expression of both *ACS8* and *ACS11* in an *acs* hextuple knockout mutant background^7^. As expected, the *acs* octuple mutant exhibited reduced levels of ethylene and displayed phenotypes similar to those of ethylene-insensitive mutants^7^. However, the octuple mutant also had reduced seed set, which was not observed in the *acs* hextuple mutant nor alleviated by ethylene treatment^7^, raising the possibility that ACC itself plays a role in reproduction.

In angiosperm reproduction, the success of the progamic phase is critically dependent on the final steps of attraction and targeting of pollen tubes to the ovules, followed by pollen tube rupture to release the sperm cells^8^. While it is generally accepted that the first task implies the secretion of small chemoattractant peptides by the embryo sac^8,9^, little is known regarding what triggers the secretion and sequential trafficking of the peptides from the synergid cells to the filiform apparatus. This secretion must be well synchronized with pollen tube progression through the pistil, with theoretical predictions suggesting that the re-direction of pollen tube growth requires adequate timing of secretion and diffusion of the attractants^10,11^.

Arguments for a role of Ca^2+^ during plant reproduction have long been proposed^12^ and live imaging has provided circumstantial evidence for a role of Ca^2+^ signaling during pollen tube/ovule interaction^13–15^. Critically, our lack of knowledge concerning the nature and regulation of the Ca^2+^ channels mediating these interactions has hampered an understanding of Ca^2+^ signaling during reproduction. Glutamate Receptor-Like (GLR) channels, which are implicated in diverse processes involving ion signaling in plants^16^, have been associated with plant reproduction from mosses to angiosperms^17–20^. However, their genetic redundancy and the lack of a comprehensive characterization of critical channel properties, such as ion and ligand selectivity^16^, have made them refractory to mechanistic association with other aspects of reproduction. Of relevance, although GLRs can be stimulated by various amino acids, the exact nature of their regulation in terms of their physiological gating ligands is unclear^10^.

Here, we analyze the basis of the *acs* octuple mutant seed set defect, finding that ACC, but not ethylene, plays a signaling role in the sporophytic tissue of the ovule effecting pollen tube attraction. Moreover, ACC promotes the ovular secretion of a known pollen tube chemoattractant, LURE1.2^21,22^, suggesting a possible basis for defective pollen tube attraction. We provide evidence that ACC is capable of gating plant GLR channels, and furthermore, is more potent than any proteinogenic amino acid in inducing a GLR-dependent cytosolic Ca^2+^ elevation in a heterologous COS-7 cell system. In ovules, ACC treatment triggers a transient Ca^2+^ elevation, and treatment with a Ca^2+^ ionophore triggers LURE1.2 secretion. These findings suggest a model in which ACC stimulates GLR-dependent Ca^2+^ elevation, which in turn promotes LURE1 secretion and pollen tube attraction.

## Results

### Fewer *acs* octuple mutant ovules are fertilized

**Fig. 1:**
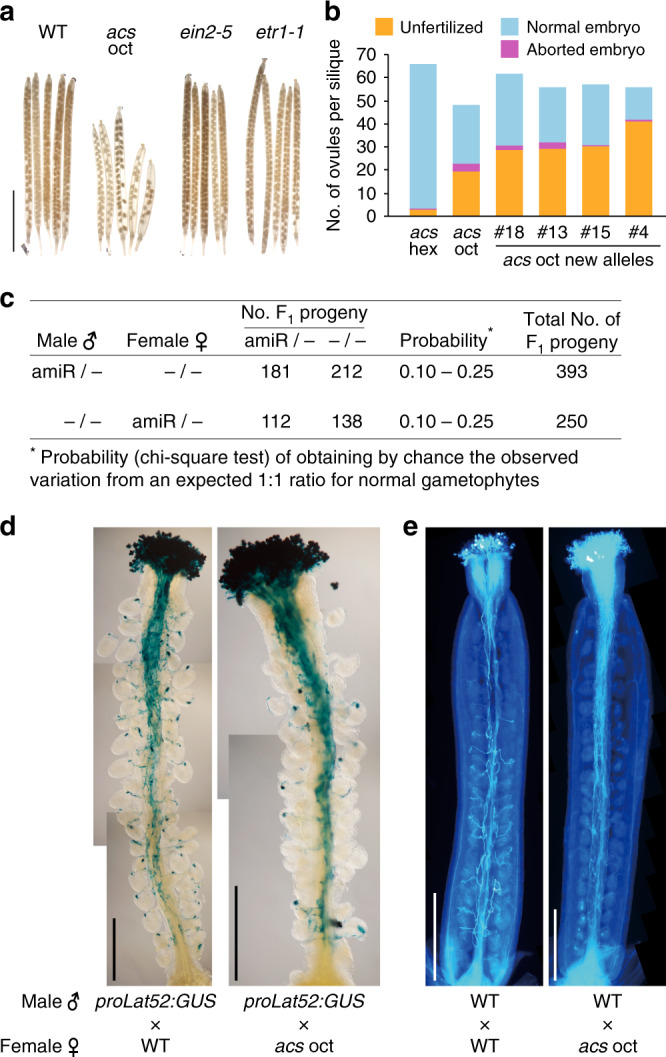
The female sporophyte of the *acs* octuple mutant is defective in pollen tube targeting. **a** Representative siliques of the *acs* octuple (oct) mutant, wild type (WT), and ethylene-insensitive mutants (*ein2-5* and *etr1-1*). Scale bar, 5 mm. **b** Average number of post-pollination ovule fates per silique in the original *acs* oct mutant^7^ and four independently generated *acs* oct mutant alleles compared to their genetic background, the *acs* hextuple (hex). *n* = 5 siliques per genotype, except *n* = 6 for Line 18. (For statistical data see Supplementary Fig. 1e–g.) **c** Reciprocal crosses between WT and the hemizygous amiRNA line. *n* = 10 crosses in each direction. **d** Representative GUS staining of WT pollen tubes expressing *LAT52:GUS* in dissected hand-pollinated pistils of the WT versus *acs* oct mutant. A dot of GUS staining within the ovule indicates fertilization by the pollen. Scale bar, 500 μm. **e** Representative images of aniline blue-stained WT pollen tubes in hand-pollinated pistils of the WT versus *acs* oct mutant. Scale bar, 500 μm.

We first confirmed that strong ethylene-insensitive mutants have normal seed set in contrast to the *acs* octuple mutant (Fig. 1a; Supplementary Fig. 1a). We then verified the reduced seed set phenotype of the *acs* octuple mutant by generating four new octuple mutant alleles, using a different amiRNA sequence to target *ACS8* and *ACS11* in the *acs* hextuple mutant background. The new octuple mutant alleles produced the same reduced ethylene levels as the original octuple mutant (Supplementary Fig. 1b). All of the octuple mutant alleles produced shorter siliques with fewer seeds (Supplementary Fig. 1c, d) and had slightly fewer ovules per pistil compared to the hextuple mutant (Fig. 1b; Supplementary Fig. 1c). The ovule number, however, did not account for the reduced seed set. By scoring the post-pollination fates of ovules, we uncovered a high percentage of unfertilized ovules per pistil (40–73%) and a low percentage of aborted embryos per pistil (0.7–7.05%) (Fig. 1b; Supplementary Fig. 1e–g). In contrast to the reported embryonic lethality (aborted embryos) in the *acs* octuple mutant^7^, these findings indicated that the underlying defect in reduced seed set occurs prior to fertilization.

For further verification of the mutant phenotype, we partially rescued the original *acs* octuple mutant by co-expressing *proACS8:ACS8m* and *proACS11:ACS11m*, each carrying silent point mutations to avoid targeting by the amiRNA. The partially rescued line had wild-type levels of ethylene (Supplementary Fig. 1b), as well as longer siliques (Supplementary Fig. 2a, b) and a higher number of fertilized ovules (Supplementary Fig. 2c–f) than the octuple mutant. In all subsequent experiments we used the original *acs* octuple mutant^7^.

### The *acs* mutant female sporophyte confers reduced seed set

Reciprocal crosses between the *acs* octuple mutant and wild type indicated that the female is responsible for the reduced seed set (Supplementary Fig. 3a–d). However, the floral organs of the *acs* octuple mutant showed no obvious morphological defects (Supplementary Fig. 3e). Hand pollination of the octuple mutant 24, 36, and 48 h after emasculating flowers at floral stage 12b^23^ did not rescue the seed number, suggesting the mutant female is not developmentally delayed (Supplementary Fig. 4a–d). To evaluate whether the defect lies with the female gametophyte or sporophyte, we carried out reciprocal crosses using *acs* hextuple mutant plants that were hemizygous for the original amiRNA. When we hand-pollinated wild-type pistils using pollen from the hemizygous plants, and vice versa, the amiRNA transgene (representing male and female *acs* octuple gametophytes, respectively) segregated 1:1 in the F_1_, indicating that neither the male or female gametophyte is responsible for the seed set defect (Fig. 1c). However, the total number of F_1_ progeny was significantly lower when wild-type pollen was used to hand-pollinate hemizygous pistils (250 F_1_ compared to 393 F_1_ for the reciprocal cross, *P* < 0.01) (Fig. 1c). These results strongly suggested that the female sporophyte is responsible for the pre-fertilization defect in the *acs* octuple mutant and indicated that the hemizygous amiRNA in the mutant is dominant, as expected. Consistent with a dominant sporophytic defect, the amiRNA segregated 3:1 (*P* = 0.25–0.50) in the self-progeny of the hemizygote.

### *acs* mutant pistils reduce pollen tube targeting of ovules

One of the most important events required for successful fertilization is the near-orthogonal change in growth trajectory of pollen tubes when exiting the transmitting tract to grow towards the ovule^8^. This altered trajectory is thought to be triggered by chemotropic factors that emanate from the embryo sac^8,9^. When we hand-pollinated the wild type versus *acs* octuple mutant using wild-type pollen expressing a pollen-specific β-glucuronidase (GUS) reporter, we observed *in planta* pollen tube targeting of 93.8% ± 3.2% of wild-type ovules (*n* = 12 pistils) compared to only 45.9% ± 15.7% of octuple mutant ovules (*n* = 19 pistils). This reduced level of targeting (Fig. 1d) is consistent with the proportion of seeds observed in the mutant. Likewise, fewer wild-type pollen tubes appeared to turn towards the ovules in octuple mutant pistils than in wild-type pistils (Fig. 1e). Pollen tubes of strong ethylene-insensitive mutants behaved similarly, turning poorly towards octuple mutant ovules (Supplementary Fig. 5a, b), thus ruling out ethylene as a chemoattractant. Conversely, an ethylene-insensitive female still attracted pollen tubes (Supplementary Fig. 5c), consistent with ethylene signaling in the female organs not playing a role in the mutant phenotype. These results suggested that the *acs* octuple mutant pistil is defective in pollen tube guidance.

### *acs* mutant ovules are defective in attracting pollen tubes

**Fig. 2:**
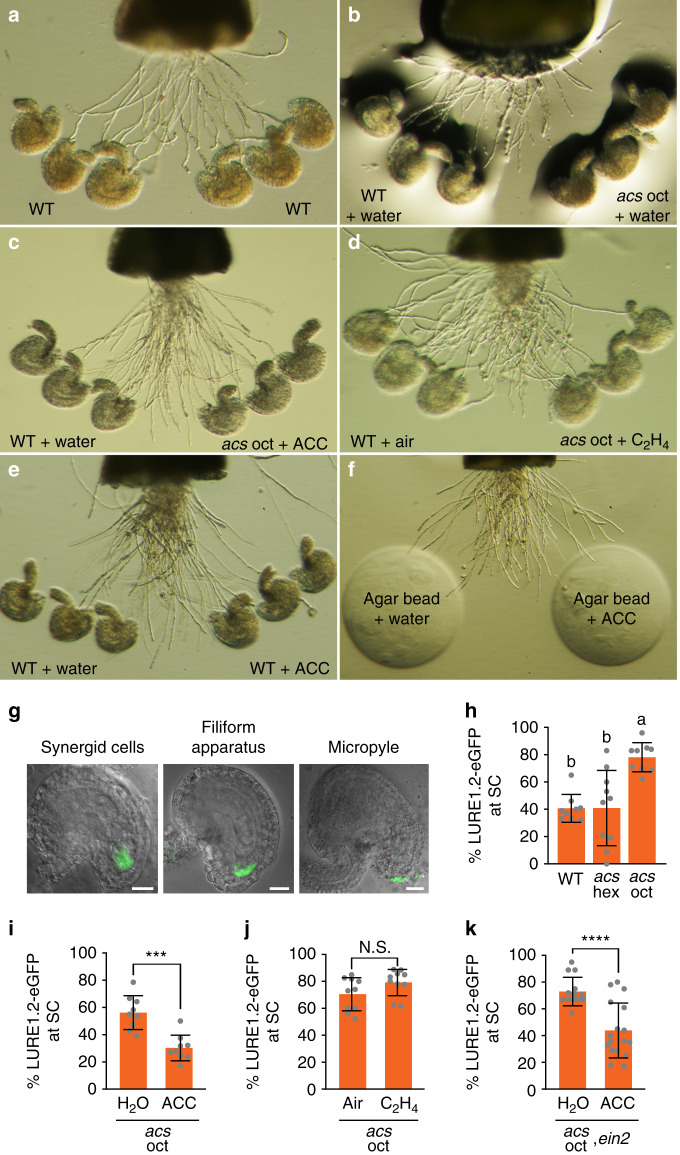
ACC has an ethylene-independent role in pollen tube attraction and LURE1.2-eGFP localization. **a**–**f** Representative images of semi-in vivo pollen tube guidance assays. Pretreatments (+water, +ACC, +C_2_H_4_) of ovules and agar beads are indicated. **g** Representative images of LURE1.2-eGFP localization in *acs* oct ovules 24 h after emasculation of stage 12c floral buds. Scale bar, 20 μm. **h-k** Average percentages of synergid cell (SC)-localized LURE1.2-eGFP in ovules per pistil (for ovules in which localization could be determined). Error bars show ± s.d. **h** No treatment; *n* = 9, 10, 9 pistils for WT, *acs* hex, *acs* oct, respectively. Different letters indicate significant difference (Welch ANOVA tests with Tamhane’s T2 multiple comparisons test *P* < 0.05, df = 2.0, *W* = 29.690). **i** Pretreatment with ACC (1 μM); *n* = 9 pistils per sample (two-tailed *t*-test, *P* = 0.0001 (***), *t* = 4.998, df = 16). **j** Pretreatment with C_2_H_4_ (10 ppm); *n* = 10 pistils per sample (two-tailed *t*-test, *P* = 0.0971 (N.S.), *t* = 1.750, df = 18). **k** Pretreatment with ACC (1 μM); *n* = 14 pistils for H_2_O, *n* = 17 pistils for ACC (two-tailed *t*-test with Welch’s correction *P* < 0.0001 (****), *t* = 5.072, df = 24.96).

To identify which parts of the mutant pistil affect pollen tube targeting, we turned to the semi-in vivo pollen tube guidance assay^24^. The assay involves placing an excised stigma-style of a hand-pollinated pistil horizontally on agar medium, allowing the pollen tubes to grow out of the cut style and along the agar surface towards excised ovules that are prearranged on the agar surface (Fig. 2a). Using this assay, we challenged wild-type pollen tubes emerging from the cut style with both wild-type and *acs* octuple mutant ovules placed on opposite sides of a midline extending from the cut style (Supplementary Fig. 6). Pollen tubes were significantly less attracted to the mutant ovules (Fig. 2b); for example, in 19 assays, a total of 227 pollen tubes (56%) turned towards the wild-type ovules, whereas 140 pollen tubes (35%) turned towards the mutant ovules (*P* < 0.01) (Table 1). There were no detectable effects on pollen tube turning when using pistils and/or pollen from the *acs* octuple mutant (Table 1). These findings indicated that the ovules of the *acs* octuple mutant are defective in pollen tube attraction.

**Table 1:** Quantification of pollen tube attraction in the semi-in vivo pollen tube guidance assay.

Fig	Pollen	Stigma-style (n)	# of pollen tubes attracted to ovules^a^				Probability^c^
			WT	*acs*	WT	Neither^b^	
2a	WT	WT (7)	93	–	96	40	0.75–0.90
–	WT	WT (19)	227	140	–	36	<0.01
2b	WT	WT (4)	76 (+H_2_O)	45 (+H_2_O)	–	27	<0.01
–	*acs*	WT (10)	118	78	–	56	<0.01
–	WT	*acs* (10)	111	76	–	45	<0.05
	*acs*	*acs* (11)	158	103	–	55	<0.01
2c	WT	WT (20)	215 (+H_2_O)	328 (+ACC)	–	151	<0.01
2d	WT	WT (13)	178 (+ambient air)	140 (+C_2_H_4_)	–	143	<0.05
–	WT	WT (8)	151 (under C_2_H_4_)	71 (under C_2_H_4_)	–	98	<0.01
2e	WT	WT (10)	215 (+H_2_O)	–	217 (+ACC)	146	0.90–0.95
			**# of pollen tubes attracted to agar beads**				
			**+H**_**2**_**O**	**+ACC**		**Neither**^**d**^	
2f	WT	WT (13)	151	138		182	0.25–0.50

^a^Ovules of the indicated genotypes were placed in the left or right regions as in Fig. 2a–e and Supplementary Fig. 6.

^b^Pollen tubes attracted to neither left or right ovules as shown in Supplementary Fig. 6.

^c^Probability (chi-square test) of obtaining by chance the observed variation from the expected 1:1 ratio (not including the data for Neither)

^d^Pollen tubes attracted to neither the water-soaked or ACC-soaked beads.

### Pollen tube attraction is rescued by ACC and not by ethylene

Notably, we rescued pollen tube attraction by pretreating *acs* octuple mutant ovules with ACC (1 μM) just prior to placing them in the semi-in vivo assay (Fig. 2c, Table 1). In contrast, pretreating the ovules with ethylene (10 ppm) did not rescue the defect (Fig. 2d, Table 1), nor did conducting the assay in the presence of ethylene (10 ppm) (Table 1). Pretreating wild-type ovules with ACC (1 μM) did not enhance their ability to attract pollen tubes (Fig. 2e, Table 1), and pollen tubes showed no preference for agar beads pre-soaked in ACC (1 μM) (Fig. 2f, Table 1), indicating a requirement for the ovule and excluding the possibility that ACC itself is a chemoattractant. From these results, we concluded that ACC, not ethylene, is an ovule-derived signal involved in mediating pollen tube attraction in *Arabidopsis*.

### *acs* octuple mutant ovules are defective in LURE1.2 secretion

Cysteine-rich LURE1 peptides are pollen tube chemoattractants sufficient to attract pollen tubes in vitro^21,22^. LURE1s are expressed in the synergid cells of the gametophyte, and prior to fertilization, they are trafficked to the filiform apparatus of the synergid cells and secreted by unknown mechanisms to the micropyle and funicular surface of the ovule^21,22^. In the *acs* octuple mutant, floral buds have normal transcript levels of *LURE1.1-1.5* (Supplementary Fig. 7a). To examine LURE1 peptide secretion, we stably expressed *proLURE1.2:LURE1.2-eGFP* in the *acs* octuple mutant. LURE1.2 has strong chemoattractant activity and is the most abundantly expressed LURE1^22^. Shown are three distinct localizations of LURE1.2-eGFP in the *acs* octuple mutant (Fig. 2g, Supplementary Fig. 7b) and in the wild type (Supplementary Fig. 7b). Interestingly, the LURE1.2-eGFP signal was more frequently observed in the synergid cells of *acs* octuple mutant ovules (imaged 24–28 h after emasculating the pistils at floral stage 12c^23^) compared to the wild type, indicating a defect in LURE1.2-eGFP trafficking or secretion to the filiform apparatus (Fig. 2h, Supplementary Fig. 7b, c). The relatively low proportion of secreted LURE1.2-eGFP, even in the wild type, could be due to the eGFP fusion. We rescued LURE1.2-eGFP secretion by pretreating octuple mutant ovules with ACC (1 μM) (Fig. 2i, Supplementary Fig. 7d), whereas we observed no difference when the ovules were pretreated with ethylene (10 ppm) (Fig. 2j, Supplementary Fig. 7e). Moreover, in an *acs* octuple mutant that was rendered ethylene-insensitive by a CRISPR/Cas9-induced deletion in the *EIN2* gene, LURE1.2-eGFP secretion was still rescued by ACC (1 μM), ruling out a role for ethylene (Fig. 2k, Supplementary Fig. 7f).

Consistent with ACC being needed in the ovule sporophytic tissue, a GUS reporter fused to the *ACS8* promoter was localized to the sporophytic tissue of the ovule (Supplementary Fig. 7g). (Expression of *ACS11*, the other gene knocked down in the octuple mutant, has not been detected in ovules^25^.) These results suggested that ACC is an inducing factor in LURE1.2 trafficking, and indicate that prevention of LURE1.2 trafficking may contribute to reduced pollen tube guidance toward the micropyle of *acs* octuple mutant ovules. ACC signaling might affect other pollen tube guidance signals^9^, including proposed long-range ovular signals that have yet to be identified^26^. Given that *acs* octuple mutants are not null mutants, a more complete loss of ACC is likely to confer additional and/or more severe phenotypes. Nevertheless, ACC in the ovule sporophytic tissue plays a unique signaling role in pollen tube guidance. This role is distinct from the function of ethylene in postfertilization synergid cell death and the pollen tube block in *Arabidopsis*^27^, and is unlike the role of ethylene in megasporogenesis in tobacco^28^.

### ACC is capable of gating plant GLR channels

**Fig. 3:**
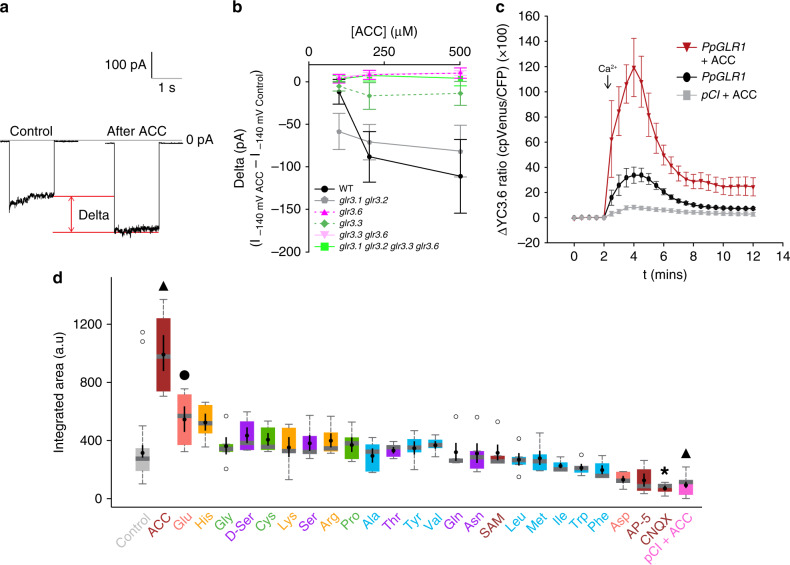
ACC stimulates GLR-dependent ion transport in *Arabidopsis* root protoplasts and mammalian COS-7 cells. **a** Macroscopic currents recorded at −140 mV under the whole-cell configuration in a WT *Arabidopsis* root protoplast show an increase (delta) after adding ACC (500 μM) to the bath solution. **b** Average currents (delta) induced by ACC (100, 200, or 500 μM) in experiments similar to that in **a** for root protoplasts of WT and *glr* knockout mutants. WT and *glr3.1*, *glr3.2*: *n* = 5 for 100, 200 μM ACC, *n* = 4 for 500 μM ACC. Quadruple mutant: *n* = 4 for 100, 200 μM ACC, *n* = 3 for 500 μM ACC. *glr3.3, glr3.6*: *n* = 4 for 100 μM ACC, *n* = 5 for 200 and 500 μM ACC. *glr3.3*: *n* = 4 for all ACC concentrations. *glr3.6*: *n* = 4 for 100 μM ACC, *n* = 6 for 200 and 500 μM ACC. Error bars show ± s.e. **c** Time course of mean cytosolic Ca^2+^ changes in COS-7 cells expressing *Pp*GLR1 monitored with YC3.6 when applying Ca^2+^ (14.5 mM; arrow) to the bath solution in the presence of ACC (500 μM; red triangles, *n* = 6) and absence of ACC (black circles, *n* = 31). Cells transfected with empty vector (pCI) were also treated with ACC (500 μM; gray squares, *n* = 10). Statistical significance was determined between the mean of each curve by two-way ANOVA (*Pp*GLR1 + ACC vs. *Pp*GLR1: *P* < 0.0001; *Pp*GLR1 + ACC vs. pCI + ACC: *P* < 0.0001; *Pp*GLR1 vs. pCI + ACC: *P* < 0.0001. F = 50.74, df = 2). Error bars show ± s.e. **d** Comparison of ACC to the 20 proteinogenic amino acids based on Ca^2+^ imaging in COS-7 cells expressing *Pp*GLR1 and YC3.6 as in **c**. Box plots show changes in cytosolic Ca^2+^ evaluated by the integrated area under the YC3.6 response curve for each amino acid (top of box is 75th percentile; bottom of box is 25th percentile; horizontal gray bar is median; gray whiskers show the maximum and minimum value within 1.5× the interquartile range) with outliers (open circles) and mean (solid black circles) ± s.e. Included are two candidate ligands, D-Serine (D-Ser) and the ACC precursor S-adenosyl-L-methionine (SAM), and two classic iGluR antagonists, 2-amino-5-phosphonovalerate (AP-5) and 6-cyano-7-nitroquinoxaline-2,3-dione disodium salt hydrate (CNQX). *n* = 31 for pCI-*Pp*GLR1 control (no amino acid added), *n* = 6 for pCI-*Pp*GLR1 + ACC, *n* = 10 for pCI (empty vector) + ACC, *n* = 5 for all other treatments. Statistical significance was determined using Dunnett’s test (solid black circle *P* < 0.1, *P < 0.05, solid black triangles *P* < 0.001).

Although not synthesized in animal tissues, ACC is a partial agonist of ionotropic glutamate receptors (iGluRs), which are ligand-gated ion channels involved in electric and Ca^2+^ signaling in neurons^29–31^. Given ACC’s signaling function in plants, we investigated whether ACC is capable of stimulating the plant homologs of iGluRs known as GLR channels. We tested whether ACC could activate GLR-dependent ion conductance using whole-cell patch-clamp on *Arabidopsis* root epidermal protoplasts, starting with the highest order *glr* mutant available (*glr3.1, glr3.2, glr3.3, glr3.6*). When treated with ACC, wild-type protoplasts displayed an increase in current amplitude, whereas those of the quadruple *glr* mutant (which displayed significantly less current without any ligand than did wild-type protoplasts: Supplementary Fig. 8a, b) were unresponsive to ACC (Fig. 3a, b). Besides this response to ACC, we confirmed these currents using the cation channel inhibitor Gd^3+^ (500 μM), which inhibited >80% of the macroscopic current, showing that the ohmic-like current recorded was not an artifactual leak but was rather induced by non-selective channels (Supplementary Fig. 8c). A *glr3.1*, *glr3.2* double mutant still responded to ACC, but the *glr3.3* and *glr3.6* single and double mutants did not respond (Fig. 3b), suggesting that the ACC-induced current is dependent on *AtGLR3.3* and *AtGLR3.6*. The lack of ACC response in *glr3.3* and *glr3.6* may be through heteromer formation, as it is well established that iGluR channels in animals are tetramers formed by homo- or heteromerization, with the specific combination of monomers having a determining role with respect to the ligand^16,32^. These results provide proof-of-principle that ACC can elicit GLR-mediated, Ca^2+^-containing currents in a physiological (root protoplast) system.

### ACC is the strongest agonist of *Pp*GLR1-driven Ca^2+^ elevation

Functional GLR ligands *in planta* remain unclear, although various candidate amino acids have been proposed^16,33,34^. The apparent promiscuity of GLR agonists arguably suggests that GLRs are adapted to functions where they process information associated with various amino acids. This view recently received support from a structural analysis of the *At*GLR3.3 ligand-binding domain^35^, raising the possibility that a preferred ligand in plants remains unaccounted for. Because *Arabidopsis* evolved twenty GLRs (more than humans), angiosperm GLRs constitute a challenging model for elucidating ligand specificity^17,33,34^. In contrast to *Arabidopsis*, the moss *Physcomitrella patens* expresses only one GLR (*Pp*GLR1) in its vegetative tissues^18^, suggesting that *Pp*GLR1 is sufficient to form functional tetra-homomeric channels. Previous characterization of *Pp*GLR1 currents by patch-clamp during heterologous COS-7 mammalian cell expression has shown them to be non-selective like those of *Arabidopsis* GLRs, with important conductance for Na^+^ and a smaller proportion of Ca^2+^ driven-currents^18^. Of relevance, the magnitude of these Ca^2+^ currents driven by *Pp*GLR1 were deemed as sufficient to sustain cytosolic Ca^2+^ elevations with enough magnitude to generate Ca^2+^-signaling events^18^.

To test ligand potency on Ca^2+^ elicitation, we thus turned to these same conditions^18^ in COS-7 cells co-expressing *Pp*GLR1 and the Ca^2+^ sensor Yellow Cameleon 3.6 (YC3.6)^36^, using Ca^2+^ imaging as a read-out. In these conditions, *Pp*GLR1 induces a cytosolic Ca^2+^ elevation when cells are exposed to extracellular Ca^2+^ in the absence of a candidate ligand, but we found that ACC strongly potentiated this response (Fig. 3c). By comparing the responses induced by ACC and all 20 proteinogenic amino acids, ACC was revealed to be the most potent activator of *Pp*GLR1-dependent cytosolic Ca^2+^ elevation (Fig. 3d, Supplementary Fig. 8d). ACC is thus a candidate ligand of the GLR channel family. The range of effects, from strongest activation (ACC) to strongest inhibition (CNQX), is also suggestive of a direct action of the amino acid on *Pp*GLR1, consistent with what is now known from the interaction of amino acids with the ligand-binding domain of *At*GLR3.3^35^.

### ACC induces GLR-mediated Ca^2+^ elevation in ovules

**Fig. 4:**
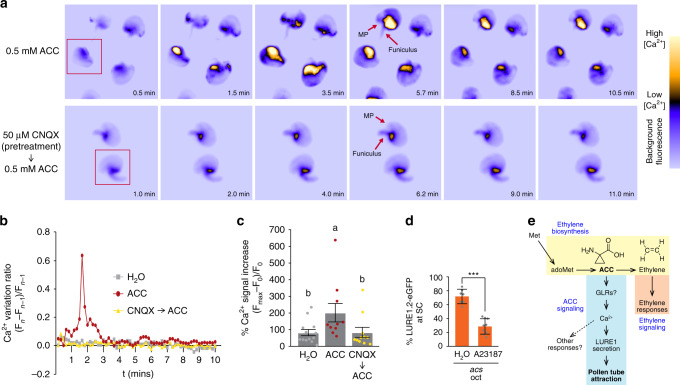
ACC stimulates CNQX-dependent Ca^2+^ elevation in ovules. **a** Representative time sequence after applying ACC (500 μM) to ovules expressing cytosolic GCaMP3. Scale reflects arbitrary units of fluorescence (see “Methods”). Top row: time sequence of transient cytosolic Ca^2+^ elevation after adding ACC. The Ca^2+^ signal first appears near the funiculus (seen from background fluorescence) and expands to the rest of the ovule. Bottom row: similar time sequence after adding ACC (500 μM) immediately after preincubation (10 min) with CNQX (50 μM). (See Supplementary Movies 1, 2 for unprocessed movies.) Red boxes indicate the ovules whose signals are plotted in **b**. **b** Ca^2+^ variation ratios for the time sequences of ovules in **a** (red boxes) and for ddH_2_O as a control. Treatments consist of ddH_2_O (gray square), ACC (red circle), and CNQX to ACC (yellow triangle). **c** Percentage of Ca^2+^ signal increase for ovules treated with 500 μM ACC (*n* = 10), 500 μM ACC (pretreated with 50 μM CNQX; *n* = 11) and ddH_2_O (*n* = 13). These data were analyzed with Kruskal–Wallis with Dunn’s post hoc test, df = 2, Kruskal–Wallis statistic = 9.062, Error bars show ± s.e. **d** Treatment with the calcium ionophore A23187 rescues LURE1.2-eGFP localization in *acs* oct ovules. Average percentage of SC-localized LURE1.2-eGFP in *acs* oct ovules per pistil (for ovules whose localization could be determined). *n* = 7 and *n* = 9 for *acs* oct pretreated with ddH_2_O and 10 μM A23187, respectively (two-tailed *t*-test, *P* < 0.0001 (****), *t* = 7.926, df = 14). Error bars show ± s.d. **e** Model of ACC signaling. Besides being the ethylene precursor, ACC induces Ca^2+^-dependent LURE1 trafficking and pollen tube attraction by the ovule through an ethylene-independent pathway that might involve GLRs.

To test whether ACC induces Ca^2+^ elevations in ovules, we imaged the cytosolic Ca^2+^ reporter GCaMP3^37^ expressed in the *ein2-5* mutant. We found that ACC induced a single, strong transient increase in Ca^2+^ levels in the ovule. This Ca^2+^ elevation forms in a sporophytic region of the ovule near the termination of the vascular bundles, then spreads to the rest of the ovule. Importantly, this increase was repressed when pretreating ovules with the iGluR antagonist CNQX (Fig. 4a–c, Supplementary Movies 1, 2). CNQX was previously shown to inhibit GLRs without discernible effect on other channels currents^16–18^, which we have now confirmed in *Pp*GLR1 (Fig. 3d). We then pretreated ovules with the Ca^2+^ ionophore A23187 (10 μM), which has been widely used to raise internal Ca^2+^ levels^38^ and has been convincingly shown to work in plant cells^39–41^. Notably, this treatment was sufficient to rescue the retention of LURE1.2-eGFP in the synergid cells of the *acs* octuple mutant ovules; we observed less LURE1.2-eGFP in the synergid cells and more in the filiform apparatus (Fig. 4d, Supplementary Fig. 9). These findings raise the possibility of a causal link between ACC-induced Ca^2+^ elevation and pollen tube attraction, in which ACC-induced Ca^2+^ elevation plays a role in LURE1 trafficking and pollen tube attraction leading to fertilization (Fig. 4e). Given our demonstration that ACC is the most potent elicitor of GLR-mediated Ca^2+^elevations, the hypothesis that these channels work as targets of ACC constitutes the most parsimonious interpretation of our data. All four of the *At*GLRs tested (Fig. 3b) are highly expressed in ovules^42^.

## Discussion

These findings support the emerging evidence of a signaling function for ACC distinct from its role as the ethylene precursor^3–7^. Seed plants typically contain micromolar levels of ACC, allowing for ACC to simultaneously serve both of these functions. Whether ACC is converted to ethylene depends on the expression and activity of ACO, and can be affected by ACC conjugation to malonyl, γ-glutamyl, or jasmonyl^43^. We have not ruled out the possibility of signaling by conjugated forms of ACC, the functions of which are unknown. Data suggest non-seed plants do not use ACC as an ethylene precursor^44^, consistent with the absence of ACO homologs in such plants^45^. Yet biosynthesis of ACC appears to have been conserved for several hundred million years prior to the emergence of seed plants^45^. Thus, ACC could have had ancient signaling roles in the plant lineage beyond angiosperms. Interestingly, ACC-induced GLR signaling fits with previous implications that GLRs were conserved in the evolution of land plant-associated cell–cell communication, which is required in fertilization^17,18^. While the triggering of post-pollination steps in ovule development has long been described^46^, we now propose a mechanism for the release of chemotropic peptides from the gametophyte controlled by the sporophytic tissue. How the stigma signal reaches the ovules is unknown, but the elicitation of electric potentials along the style following compatible pollination have been reported^47–49^, and such long-range electric communication between organs has been recently associated with GLR function^50,51^. Interestingly, the propagation of these electric potentials occur along the phloem tissue, and our data suggest that ACC-induced Ca^2+^ signals in the ovule lie at the termination of the vascular bundles in the phloem.

## Methods

### Plant materials and general growth conditions

*Arabidopsis thaliana* ecotype Columbia (Col-0) was the wild type. The *acs* hextuple (*acs2-1, acs4-1, acs5-2, acs6-1, acs7-1, acs9-1*) and octuple mutants were obtained from the Arabidopsis Biological Resource Center (ABRC) and confirmed by genotyping; *acs2-1*, *acs4-1*, *acs5-2*, *acs6-1*, *acs7-1*, and *acs9-1* alleles were genotyped as described^7^ using T-DNA left border primer “TL-3” (5′ GTACATTAAAAACGTCCGCAATGTG-3′) (except for *acs7-1*), and the amiRNA transgene, (*amiR*) was genotyped using primers shown in Supplementary Table S1. Except where specified, all analyses of the octuple mutant were carried out using the previously published homozygous *acs* octuple mutant^7^. A transgenic wild-type line expressing the β-glucuronidase (GUS) reporter from the pollen-specific *LAT52* promoter (*proLAT52:GUS*)^52^ was a gift from Ravishankar Palanivelu (University of Arizona, Tuscon). Double and quadruple *glr* mutants were from Edward Farmer (University of Lausanne, Switzerland). The *eto1, eol1, eol2* mutant was from Dr. Bram Van de Poel (KU Leuven, Belgium).

To grow plants, surface-sterilized seeds were cold stratified for 3 days on 0.8% agar medium containing 1x Murashige and Skoog salts (Caisson Labs), then germinated under 16- or 24-h light (~120 mol m^−2^ s^−1^) at 22 °C except where noted. Ten-day-old seedlings were transferred to soil (2 parts Fafard Fine Germination Mix:1 part Vermiculite) and grown in chambers under 16-h light/8-h dark at 22 °C. Relative humidity was maintained at 45–70%.

### Seed number and silique length measurements

Plants of each genotype were grown side-by-side under the same conditions. Siliques at the mature green stage (7–10 days post-pollination) were collected from the middle of the main stem, excluding the first 4–5 siliques and the last several siliques. To count seeds, the carpel walls were cleared by incubating the siliques in 95% EtOH for about 1 week, then photographed under a stereoscopic zoom microscope (Nikon SMZ1000). The number of seeds per silique was counted based on the images, and silique length was determined using ImageJ^53^.

### New *acs octuple* mutant alleles

Plasmid pRS300^54^ was used as a PCR template to create a new artificial microRNA (*amiR*) distinct from that used in Tsuchisaka et al.^7^. We PCR-amplified three DNA fragments, using primers A + IV, III + II and I + B shown in Supplementary Table S1, as described^7^, and cloned the fragments by Gibson assembly into *Spe*I/*Kpn*I-digested binary vector pMDC32^55^. The construct was transformed into the *acs* hextuple mutant by the floral dip method^56^ using *Agrobacterium tumefaciens* strain *GV3101 (pMP90)*^57^. T_1_ transformants were selected on hygromycin B (15 μg/mL) as described^58^ and confirmed by PCR-genotyping of both the hygromycin resistance gene and the *amiR* sequence, using primers shown in Supplementary Table S1. The T_1_ plants were used for determining ovule fates. Homozygous T_3_ progeny were used for ethylene measurements.

### Complementation of the *acs* octuple mutant

We complemented the original *acs* octuple mutant^7^ by expressing genomic versions of the previously-described *Arabidopsis ACS8m* and *ACS11m* cDNA sequences^7^ (mutated to prevent targeting by the *amiR*). Instead of the CaMV 35S promoter, we used the native promoter regions of *ACS8* and *ACS11*, 2544 bp and 2755 bp upstream of each start codon, respectively. For each gene, the wild-type genomic sequence spanning the upstream region to the stop codon was amplified in two overlapping fragments using primers shown in Supplementary Table S1. The altered *amiR* target site was carried by the PCR primers that generated the overlapping fragments. The fragments, *proACS8:ASC8m* and *proACS11:ASC11m*, were each separately cloned by Gibson assembly into *Eco*RI-digested Gateway entry vector pENTR2B and confirmed by DNA sequencing, before their transfer by Gateway cloning into binary vectors pEarlyGate303 and pMDC99 (ABRC), respectively. (*Sac*I/*Spe*I-digested pMDC99 was first modified by Gibson assembly to carry an OCS terminator sequence that was PCR-amplified from pEarleyGate103 using primers shown in Supplementary Table S1). Finally, *proACS8:ACS8m* was subcloned from pEarlygate303 into pMDC99-*proACS11:ACS11m* by ligation into *BamH*I and *Sbf*I sites. The resulting plasmid, carrying both *proACS8:ACS8m* and *proACS11:ACS11m*, was stably transformed into the *acs* octuple mutant as described above. Transgenic plants were confirmed by PCR-genotyping of both the hygromycin resistance gene and the transgene using primers shown in Supplementary Table S1.

### Ethylene measurement

Seeds were surface sterilized, then stratified for 3 days at 4 °C, then grown on a sterile filter paper on top of 1X MS medium (0.8% agar) for 9 days under light (16 h/8 h) at 22 °C. Approximately 20 seedlings of each genotype (six biological replicates per genotype) were then placed in 300 μL liquid MS medium in 4-mL vials fitted with septa-containing airtight caps (Fisher, cat# 03-391-22). After incubating the vials under light (16 h/8 h) at 22 °C for 48 h, we assayed the ethylene concentration in the vial headspace by gas chromatography (Shimadzu GC-2010 Plus). Seedlings were then removed from the vials, blotted briefly on a kimwipe, then weighed. We confirmed there is no ethylene peak for medium in the vial without seedlings. Transgenic lines were homozygous.

### Ovule fate determination

Plants of each genotype were grown side-by-side under the same conditions. Siliques at the mature green stage (7–10 days post-pollination) were collected from the middle of the main stem, excluding the first 4–5 siliques and the last several siliques. Siliques were placed on double-side tape and the ovary walls were removed using a 27.5-gauge needle under a dissection microscope as described^59^. Ovule fates were scored in the T_1_ generation for the new *acs* octuple mutants, since the amiRNA responsible for generating the octuple mutant was dominant. Fertilized ovules that developed normal-looking embryos were scored as “normal embryos”. Embryos that were pale green, white, or brown and somewhat flattened were scored as “aborted embryos”. Unfertilized ovules, which were very small and wrinkled, were scored as “unfertilized ovules”. Images were obtained using a stereoscopic zoom microscope (Nikon SMZ1000).

### Reciprocal crosses and pollination time course

For reciprocal crosses in Supplementary Fig. 3, we hand-crossed wild-type pistils using *acs* octuple mutant pollen, and vice versa. As controls, each genotype was hand-pollinated to itself. For subsequent reciprocal crosses in Fig. 2, the *acs* hextuple mutant (female) was crossed with the original *acs* octuple mutant^7^ (male) to generate F_1_ plants homozygous for the *acs* hextuple knockout insertions and hemizygous for the *amiR* transgene^7^ (responsible for creating the *acs* octuple mutant in the *acs* hextuple background). Reciprocal crosses were then carried out between the F_1_ and wild type, and the resulting progeny were genotyped for *amiR* using primers listed in Supplementary Table S1 to score segregation of the gentamicin resistance gene carried by the *amiR* transgene. Inheritance of the *amiR* by half the progeny indicated the absence of a gametophytic defect. (A significant reduction in *amiR* transmission would have indicated a gametophytic defect). For the pollination time course, flowers were emasculated at stage 12b^23^, then hand pollinated with wild-type pollen after 24, 36, and 48 h.

### Examination of flower/ovule morphology

Plants of each genotype were grown side-by-side under the same conditions. Flowers at stage 12b (pre-pollination)^23^ and stage 14 (post-pollination)^23^ were collected from the middle of the main stem, excluding the first four to five flowers and the last several flowers. Some or all of the outer organs were removed. To examine ovule morphology, stage 12b flowers were emasculated and pistils were dissected two days later as described above for ovule fate determination. Images were obtained using a stereoscopic zoom microscope (Nikon SMZ1000).

### Pollen tube visualization using *proLAT52:GUS*

Wild-type and *acs* octuple mutant flowers were emasculated at floral stage 12b^23^, then hand-pollinated 24 h later using wild-type pollen expressing *proLAT52:GUS*. For GUS staining, pistils were harvested 22 h after pollination, and ovary walls were removed as described above for ovule fate determination. Dissected pistils were immediately placed in 80% acetone for 4 h then incubated in GUS staining solution (5 mM potassium ferrocyanide, 5 mM potassium ferricyanide, 50 mM NaPO_4_, pH 7, 0.5 mg/mL 5-bromo-4-chloro-3-indolyl-β-D-glucuronic acid) at 37 °C overnight in the dark in a humid chamber. The GUS stain was visualized using a Zeiss Axioskop 50 microscope, and images were captured using a Sony ILCE-7RM3 camera at ×10 magnification. Composite images were created by pairwise stitching of individual images using Photoshop CS5 (Adobe).

### Pollen tubes staining with aniline blue

Flowers were emasculated at stage 12b^11^ and the pistils were hand-pollinated 24 h later. Pistils were detached 18 h after pollination and fixed in ethanol:acetic acid (3:1) for at least 2 h at room temperature, then rehydrated in an ethanol series (70% EtOH, 50% EtOH, 30% EtOH, then ddH_2_O for 10 min each). The pistils were then incubated in 8 M NaOH overnight at room temperature, washed with distilled water for 10 min, then stained overnight at room temperature with decolorized aniline blue 0.1 % (w/v) in the dark as described^60^. Decolorized aniline blue solution was made by preparing 0.1% (w/v) aniline blue in 108 mM K_3_PO_4_ (pH ~ 11). After storage at 4 °C overnight, the solution was filtered through filter paper lined funnel with a teaspoon of active carbon powder. Pistils were mounted on slides in 80 μL aniline blue stain solution with a cover slip, then imaged using a DeltaVision Elite Deconvolution/TIRF microscope with a 10x objective using an excitation wavelength of 420–490 nm and emission wavelength of 510 nm. Composite images were created using the ImageJ pairwise stitching plugin^61^ to join individual images. We examined at least 15 pistils per genotype.

### Semi-in vivo pollen tube guidance assay

The semi-in vivo pollen tube guidance assay was performed as described^24^. In brief, after hand-pollination, the pistil was cut horizontally at the junction of style and ovary, then placed on pollen tube growth medium (18% Sucrose; 0.01% Boric acid; 1 mM CaCl_2_; 1 mM Ca(NO_3_)_2_; 1 mM MgSO_4_; 0.5% Noble agar (Difco)) in a small petri dish (35 mm diameter). Next, ovules were excised from pistils and immediately placed on the medium with micropylar ends facing the cut style. For “ovule competition”, wild-type and mutant ovules (harvested 24 h after emasculation at floral stage 12b^23^) were arranged on the left/right or right/left sides with respect to the cut style. Each agar plate (with the lid on) was incubated on a water-soaked paper towel inside a larger (150 mm diameter) petri dish (with the lid on) in an airtight food storage container that was also lined with a water-soaked paper towel. After ~4 h at room temperature, pollen tubes were viewed under a Leica M205 FA microscope and images captured using a SONY SLT-A55V camera.

Evaluation of micropylar guidance of pollen tubes was based on separate regions beneath the cut style. As shown in Supplementary Fig. 6, we defined the left and right ovule regions as the areas extending from the center of the cut style to between the micropyle of the leftmost or rightmost ovule and the micropyle of the central-most ovule on the left or right, respectively. All the pollen tube tips in the left region (or right) were scored as turning to the ovules on the left (or right). Pollen tube tips between the left and right regions were regarded as turning to neither region. Pollen tube tips at either the far left or far right of the defined regions were considered out of range and were not included in the total pollen tube number.

For ACC pretreatment, we dissected pistils 24 h after emasculation at floral stage 12b^23^ as described above for ovule fate determination, and to each dissected pistil we added 1.0 μL 1 μM ACC, which covered all the ovules in the pistil. Immediately after absorbing the ACC solution (5–10 min), ovules were arranged on the agar medium taking care not to let the ovules dry out. Pretreatment with 1.0 μL sterile ddH_2_O was used as a control. For ethylene pretreatment, the dissected pistil was transferred to semi-in vivo agar medium in a small petri dish, and the dish (with its lid partially off) was placed on a water-soaked paper towel in an airtight 1.0 L Mason jar fitted with a septum in its lid. Ethylene gas was injected through the septum to provide a final concentration of 10 ppm in the jar. Injection of ambient air was the control. After 3 h at room temperature, the ovules were placed in the semi-in vivo assay as above. For continuous ethylene treatment, we conducted the entire assay under ethylene by placing the agar plate containing the stigma-style and ovules into the Mason jar, injecting ethylene to a final concentration of 10 ppm and incubating for ~4 h before imaging.

To test whether ACC functions as a pollen tube chemoattractant, we used 6% Sepharose beads (200–300 μm) (Colloidal Science Solutions) in place of ovules. Beads were prewashed five times (5 min each) with sterile ddH_2_O, then immersed in either 1 μM ACC or ddH_2_O for 30 min prior to positioning a bead on the agar medium on either side below, and equidistant to, the cut style.

### qPCR of LURE1s

RNA was extracted from stage 12b flowers of the *acs* hextuple and octuple mutants using SpectrumTM Plant Total RNA Kit (Sigma), then reverse-transcribed using iScript™ cDNA Synthesis Kit (Bio-Rad). qPCR was performed on a Bio-Rad CFX96 system using iTaq^TM^ Universal SYBR Green Supermix (Bio-Rad), using a single primer pair (Supplementary Table S1) to amplify a conserved sequence in all five LURE1.1-1.5 genes. Relative expression was calculated using the 2^-ΔΔCT^ method^62^ normalizing to *TUBULIN5* (AT1G20010.1) using primers shown in ref. ^20^. We carried out 12 biological replicates per genotype, and three technical replicates per biological replicate.

### *proLURE1.2:LURE1.2-eGFP* construct and localization

To generate the plasmid backbone, binary vector pEarleyGate103 (ABRC) was digested with *Mlu*I and *Pac*I, which removed the CaMV 35S promoter and *GFP* sequence. The following DNA fragments were PCR-amplified using primers shown in Supplementary Table S1: (a) a fragment spanning the *Mlu*I site to just before the start of the CaMV 35S promoter amplified from pEarleyGate103, (b) a 658-bp fragment containing the *LURE1.2* promoter/coding region amplified from wild-type *Arabidopsis* genomic DNA, and (c) the *eGFP* coding sequence amplified from plasmid pK7FWG2^63^ (ABRC). After gel-purification, the three fragments and plasmid backbone were combined by Gibson assembly.

We next replaced the Basta-resistance gene in the resulting plasmid with a hygromycin-resistance gene, as the *acs* octuple mutant is already Basta-resistant. We removed the Basta-resistance gene by *Kpn*I and *Cla*I, and a hygromycin-resistance gene fragment was PCR-amplified from pMDC99 using primers listed in Supplementary Table S1. Fragments were combined by Gibson assembly creating the final plasmid *proLURE1.2:LURE1.2-eGFP*. Plants were transformed and homozygous lines obtained as described for the new *acs octuple* mutant alleles.

For eGFP visualization, flowers of hygromycin-resistant T_2_ or homozygous T_3_ plants were emasculated at floral stage 12c^23^, and pistils were harvested 24 h later as described^64^. Pistils were dissected and mounted on microscope slides as described^59^. For pretreatment with ACC (1 μM), ethylene (10 ppm), or A23187 (10 μM), ovules were treated as described for the semi-in vivo pollen tube guidance assay, except they were incubated for 2.5–3.5 h. After treatment, pistils were transferred to slides and mounted with Fluoromount-G^®^ (SouthernBiotech) and coverslips. eGFP fluorescence was visualized under a Zeiss LSM 710 laser scanning confocal microscope coupled with ×40 oil-immersion objective, exciting eGFP with a 488-nm argon laser. The *Z*-axis focus was adjusted during observation.

### Knockout of *ein2* in the *acs* octuple mutant

Binary vector V3^20^, carrying eGFP-tagged oleosin to screen for transformants, was digested with *Spe*I/*Eco*RI and ligated to a 6275 bp *Spe*I/*Eco*RI fragment (containing the Ec1.2enEC1.1 promoter, zCas9 and RBCS terminator) of plasmid p425-pHEE401E^65^. The resulting plasmid was digested with *Kpn*I/*Spe*I and combined by Gibson assembly with a Gateway destination cassette (PCR-amplified from a modified pHEE401E^20^ template using primers shown in Supplementary Table S1) to create destination vector p568.

To create the *EIN2* CRISPR/Cas9 transformation vector, we generated two guide RNAs shown in Supplementary Table S1) that targeted different locations in the *EIN2* sequence. Four overlapping fragments were PCR-amplified from pCAMBIA1302-Cas9-sgRNA^66^ using the primers shown in Supplementary Table S1. The fragments were cloned by Gibson assembly into *Sal*I/*Xho*I-digested pENTR2B (Invitrogen), creating an *EIN2* guide plasmid (U6-26pro:gRNA1-scaffold-U6-26 terminator and U6-26pro:gRNA2-scaffold-U6-26 terminator), which we confirmed by DNA sequencing. The construct was transferred into p568 by Gateway cloning.

The final construct was transformed into the *acs* octuple mutant as described above, except the *Agrobacterium* carried the pSoup^67^ helper plasmid. T_1_ seeds were identified based on seed coat fluorescence, and PCR-based genotyping was used to identify deletions in the *EIN2* sequence using primers shown in Supplementary Table S1. In the T_2_ generation, we obtained one plant that was heterozygous for a 407-bp deletion, and we identified a homozygous mutant via PCR-screening of T_3_ progeny that lacked zCas9 (the seeds were non-fluorescent). The homozygous line was confirmed by assaying for absence of the ethylene response in dark-grown seedlings^1^, and then transformed with *proLURE1.2:LURE1.2-eGFP* as described above. T_1_ plants were analyzed.

### proACS8:GUS

The *ACS8:GUS* reporter gene^25^ (ABRC, #CD3-721) was stably transformed into wild-type *Arabidopsis* as above, and transformants were selected on gentamycin (100 μg/mL). Stage 12b flowers were emasculated and ovary walls were removed 24 h later as described^59^. Dissected pistils were stained and imaged for GUS activity as described for *proLAT52:GUS*. We analyzed ten T_1_ plants, and six showed the same expression pattern shown in Supplementary Fig. 7g.

### Patch-clamp of *Arabidopsis* protoplasts

Roots were collected from 14-day-old *Arabidopsis* seedlings grown on MS agar medium under long day (16 h) at 19 °C with the plates incubated vertically. The root digestion solution, which was prepared 12 h prior to use and stored at 4 °C, consisted of 1.5% w/v cellulase R10 (Omozuka), 0.4% w/v pectolyase Y23 (Omozuka), 1.5% cellulozyme (Fisher), 0.4 M D-Mannitol, 20 mM MES (pH 5.7, Tris HCl) and 20 mM KCl). The solution was heated for 10 min at 55 °C, and then BSA and CaCl_2_ were added to a final concentration of 0.1% BSA and 10 mM CaCl_2_. Roots were incubated in this solution for 1 h at 30 °C with agitation. Protoplasts were centrifuged and washed in two successive rounds with patch-clamp bathing solution (10 mM NaCl, 20 mM CaCl_2_, 90 mM NMDG-Cl and 10 mM Bis-Tris propane, pH 6.5 (MES)). The pipette solution contained 140 mM NaCl, 3 mM MgCl_2_, 5 mM EGTA, and 10 mM Bis-Tris propane pH 7.2 (HEPES). Solutions were adjusted to 400 mOs mol kg^−1^ with D-mannitol. Protoplasts were stored on ice up to 3 h prior to experimentation. Pipettes were pulled with a P97 puller (Sutter Instrument). Their resistance was 10–20 Mohm. Currents were recorded after establishing the whole-cell configuration^68^, filtered at 1–2 kHz with a sampling frequency of 2–4 kHz using an Axopatch 200 A amplifier, digidata 1200 series interface and Clampfit6 software (Molecular Devices). After entering the whole-cell configuration, we waited 10 min for the pipette solution to diffuse into the protoplast and the current to stabilize. The voltage protocol consisted of 1.6 s-long pulses from −140 mV to + 60 mV (20 mV steps) both before, and 5 min after, adding either ACC or GdCl_3_ to the bath solution.

### Ca^2+^ imaging in COS-7 cells

COS-7 cells (ATCC and Sigma-Aldrich) were maintained at 37 °C and 5% CO_2_ in Dulbecco’s Modified Eagle’s Medium, supplemented with 5% fetal bovine serum and 1% penicillin/streptomycin (Gibco), and transfected at low passage (*P* < 7). COS-7 cells were plated at a density of 50% confluence in 35-mm diameter dishes and transfected using FugeneHD (Promega) as specified by the supplier. For Ca^2+^ imaging, pCI (0.6 µg or 1 µg) or pCI-*PpGLR1*^28^ (1 µg) was co-transfected with pEF1-YC3.6 (0.5 µg). COS-7 cells were transferred to new petri dishes 24 h after transfection (by trypsin treatment) at low density.

For Ca^2+^ imaging, COS-7 cells expressing YC3.6 were washed in a Ca^2+^-free solution (1 mM EGTA, 10 mM Bis-Tris propane buffered to pH 7.3 (HEPES) and set to 335 mosmol kg^−1^ with D-mannitol). Cells were imaged in the Ca^2+^-free solution for 2.5 min before the addition of Ca^2+^ to a final concentration of 14.5 mM. Imaging was performed at room temperature using a DeltaVision Elite Deconvolution/TIRF microscope system (Olympus inverted IX-71) under a 60× lens (1.2NA UPLSAPO water lens/WD 0.28 mm) as described^69^. A xenon lamp from the DeltaVision system was used with a CFP excitation filter (438–424 nm). Two simultaneous emission records were captured: YFP emission (548–522 nm) and CFP emission (475–424 nm). To minimize bleaching, the laser was set to 2%. YFP and CFP imaging were recorded with 0.3 s exposure time. Time-lapse acquisition was performed with a sampling interval of 30 s. Images were processed using ImageJ^53^. Ratios were obtained after background subtraction and signal clipping using the “Ratio-plus” plug-in for ImageJ as described^69^. In the ligand screening experiments (Fig. 3d and Supplementary Fig. 8d), values were multiplied by a factor of one hundred. The signal of each channel was averaged in a circle in the middle of the cell (with 100–200 pixel diameter depending on the size of the cell). The YFP/CFP ratio was obtained by dividing the emission recorded for YFP (548–522 nm) by the one recorded for CFP (475–424 nm). No significant bleaching or ratio drift was observed in our experimental conditions. To standardize YC3.6 responses, the baseline values (five points prior to Ca^2+^ application) were averaged and then subtracted from each measurement recorded. The integrated area under the curve was calculated in SigmaPlot 11.0 (Systat Software Inc) by drawing a best-fit line and using the built in macro “Area Below Curves”.

### Ca^2+^ imaging in *ein2-5* ovules

We crossed a cytosolic GCaMP3 reporter in the wild type (gift from Simon Gilroy, University of Wisconsin, Madison) into the *ein2-5* mutant and identified double homozygotes in the F_3_ based on ethylene insensitivity and kanamycin resistance (50 μg/mL). Flowers were emasculated at Stage 12c, and 24 h later the ovules were cut from dissected pistils and placed on a thin layer of agar medium created by spreading 100 µL of pollen germination medium^24^ over the entire coverslip of a glass bottom petri dish (MatTek, P35G-1.0-14-C).

Ca^2+^ signals were visualized under a Nikon ECLIPSE microscope, using dry PlanApo lenses of either 20×(NA0.75) or 40× (NA1.0). Wavelength was controlled by a Prior Lumen 200 Pro Fluorescence Illumination System, with a GFP filter, and images were captured by a iXON3 ANDOR camera using Micro-Manager 1.4.16. Prior to treatments, we imaged ovules every 10 s for 2 min. For treatments, 5 μL of ACC (500 μM), CNQX (50 μM), or sterile ddH_2_O was added to the surface of the germination medium covering all of the ovules, and the microscope was immediately refocused. We then imaged the ovules every 10 s for 10 min. For the CNQX-treated samples, we subsequently added ACC (500 μM), and continued imaging every 10 s for 10 min. We imaged 10 ovules treated with ACC, 11 ovules treated with ACC after CNQX pretreatment, and 13 ovules treated with ddH_2_O.

We quantified Ca^2+^ signal intensity using ImageJ^70^. We selected the entire ovule as the “region of interest (ROI)” and quantified ROI fluorescence by selecting “Mean” under “Set measurements”. In the ROI menu, we chose “more” and “multi measure” to obtain the mean pixel value across all time points. We subtracted background fluorescence from each ovule pixel value. Background was determined by measuring the pixel value of three blank areas, each of similar size and shape as an ovule, and then averaging the values. Further processing was made by normalizing sequences to background values, and applying the ICA LUT for visualization.

The relative Ca^2+^ variance ratio time course (Fig. 4b) was calculated as (*F*_*n*_ − *F*_*n*−1_)/*F*_*n*−1_ where *F*_n_ is the fluorescence value at time point *n*, and *F*_*n*−1_ is the value at one time point prior. The percentage of Ca^2+^ signal increase (Fig. 4c) was calculated as (*F*_max_ − *F*_0_)/*F*_0_ where *F*_0_ is the value for the first frame in the time series and *F*_max_ is the maximum value in the series.

### Statistical analyses

All measurements were taken from distinct samples. All statistical analyses were performed using Prism (8.0.1, GraphPad) or SigmaPlot (v11.0, Systat Software Inc). For comparisons between two groups, we used the *t*-test (two tailed) for populations having a normal (Gaussian) distribution when both groups had the same SD. When the two populations did not have the same SD, we used the *t*-test with Welch’s correction (two tailed). For populations lacking a normal (Gaussian) distribution, we used two nonparamatric tests: the Mann–Whitney test to compare ranks and the Kolmogorov–Smirnov test to compare the cumulative distribution. For comparisons among multiple groups, we used one-way ANOVA with Tukey’s HSD post hoc test for populations that have normality of residuals and the same SD. When SDs were unequal, we used the Welch ANOVA tests with Tamhane’s T2 post hoc test. For data lacking a normal distribution of residuals, we used the nonparametric Kruskal–Wallis test with Dunn’s post hoc test. For Fig. 3a, b and Supplementary Figs. 8a, b, we used ANOVA. For Fig. 3d and Supplementary Fig. 8d, we used a Dunnett’s test for comparison of multiple experimental treatments to a single control. For Fig. 3c, we used the RM two-way ANOVA with Geisser-Greenhouse correction with Tukey’s HSD post hoc test to compare the mean value for each of the whole curve.

### Reporting summary

Further information on research design is available in the Nature Research Reporting Summary linked to this article.

## Supplementary information

Supplementary Information

Descriptions of Additional Supplementary Files

Supplementary Movie 1

Supplementary Movie 2

Reporting Summary

## Data Availability

Source data are provided with this paper as a Source Data file. Other data and biological materials are available from the corresponding authors upon reasonable request. Source data are provided with this paper.

## Source data

Source Data

## References

[1] Abeles, F. B. M., Morgan, P. W. & Saltveit, M. E. *Ethylene in Plant Biology* (Academic, 1992).

[2] Adams DO, Yang SF (1979). Ethylene biosynthesis—identification of 1-aminocyclopropane-1-carboxylic acid as an intermediate in the conversion of methionine to ethylene. Proc. Natl Acad. Sci. U.S.A..

[3] Vanderstraeten L, Depaepe T, Bertrand S, Van Der Straeten D (2019). The ethylene precursor ACC affects early vegetative development independently of ethylene signaling. Front. Plant Sci..

[4] Tsang DL, Edmond C, Harrington JL, Nuhse TS (2011). Cell wall integrity controls root elongation via a general 1-aminocyclopropane-1-carboxylic acid-dependent, ethylene-independent pathway. Plant Physiol..

[5] Xu SL, Rahman A, Baskin TI, Kieber JJ (2008). Two leucine-rich repeat receptor kinases mediate signaling, linking cell wall biosynthesis and ACC synthase in Arabidopsis. Plant Cell.

[6] Yin J (2019). Aminocyclopropane-1-carboxylic acid is a key regulator of guard mother cell terminal division in *Arabidopsis thaliana*. J. Exp. Bot..

[7] Tsuchisaka A (2009). A combinatorial interplay among the 1-aminocyclopropane-1-carboxylate isoforms regulates ethylene biosynthesis in *Arabidopsis thaliana*. Genetics.

[8] Johnson MA, Harper JF, Palanivelu R (2019). A fruitful journey: pollen tube navigation from germination to fertilization. Annu. Rev. Plant Biol..

[9] Zhong S (2019). Cysteine-rich peptides promote interspecific genetic isolation in Arabidopsis. Science.

[10] Stewman SF (2010). Mechanistic insights from a quantitative analysis of pollen tube guidance. BMC Plant Biol..

[11] Feijó JA (2010). The mathematics of sexual attraction. J. Biol..

[12] Ge LL, Tian HQ, Russell SD (2007). Calcium function and distribution during fertilization in angiosperms. Am. J. Bot..

[13] Ngo QA, Vogler H, Lituie DS, Nestorova A, Grossniklaus U (2014). A calcium dialog mediated by the FERONIA signal transduction pathway controls plant sperm delivery. Dev. Cell..

[14] Denninger P (2014). Male-female communication triggers calcium signatures during fertilization in Arabidopsis. Nat. Commun..

[15] Hamamura Y (2014). Live imaging of calcium spikes during double fertilization in Arabidopsis. Nat. Commun..

[16] Wudick MM, Michard E, Nunes CO, Feijó JA (2018). Comparing plant and animal glutamate receptors: common traits but different fates?. J. Exp. Bot..

[17] Michard E (2011). GLUTAMATE RECEPTOR-LIKE genes form Ca^2+^ channels in pollen tubes and are regulated by pistil D-serine. Science.

[18] Ortiz-Ramirez C (2017). GLUTAMATE RECEPTOR-LIKE channels are essential for chemotaxis and reproduction in mosses. Nature.

[19] Michard E, Simon AA, Tavares B, Wudick MM, Feijó JA (2017). Signaling with ions: the keystone for apical cell growth and morphogenesis in pollen tubes. Plant Physiol..

[20] Wudick MM (2018). CORNICHON sorting and regulation of GLR channels underlie pollen tube Ca^2+^ homeostasis. Science.

[21] Okuda S (2009). Defensin-like polypeptide LUREs are pollen tube attractants secreted from synergid cells. Nature.

[22] Takeuchi H, Higashiyama T (2012). A species-specific cluster of defensin-like genes encodes diffusible pollen tube attractants in *Arabidopsis*. PLoS Biol..

[23] Christensen CA, King EJ, Jordan JR, Drews GN (1997). Megagametogenesis in Arabidopsis wild type and the Gf mutant. Sex. Plant Reprod..

[24] Palanivelu R, Preuss D (2006). Distinct short-range ovule signals attract or repel *Arabidopsis thaliana* pollen tubes *in vitro*. BMC Plant Biol..

[25] Tsuchisaka A, Theologis A (2004). Unique and overlapping expression patterns among the Arabidopsis 1-amino-cyclopropane-1-carboxylate synthase gene family members. Plant Physiol..

[26] Mizuta Y, Higashiyama T (2018). Chemical signaling for pollen tube guidance at a glance. J. Cell Sci..

[27] Völz R, Heydlauff J, Ripper D, von Lyncker L, Gross-Hardt R (2013). Ethylene signaling is required for synergid degeneration and the establishment of a pollen tube block. Dev. Cell.

[28] De Martinis D, Mariani C (1999). Silencing gene expression of the ethylene-forming enzyme results in a reversible inhibition of ovule development in transgenic tobacco plants. Plant Cell.

[29] Inanobe A, Furukawa H, Gouaux E (2005). Mechanism of partial agonist action at the NR1 subunit of NMDA receptors. Neuron.

[30] Yao Y, Harrison CB, Freddolino PL, Schulten K, Mayer ML (2008). Molecular mechanism of ligand recognition by NR3 subtype glutamate receptors. EMBO J..

[31] Kristensen AS (2016). Pharmacology and structural analysis of ligand binding to the orthosteric site of glutamate-like GluD2 receptors. Mol. Pharmacol..

[32] Traynelis SF (2010). Glutamate receptor ion channels: structure, regulation, and function. Pharmacol. Rev..

[33] Vincill ED, Bieck AM, Spalding EP (2012). Ca^2+^ conduction by an amino acid-gated ion channel related to glutamate receptors. Plant Physiol..

[34] Tapken D (2013). A plant homolog of animal glutamate receptors is an ion channel gated by multiple hydrophobic amino acids. Sci. Signal..

[35] Alfieri A (2020). The structural bases for agonist diversity in an Arabidopsis thaliana glutamate receptor-like channel. Proc. Natl Acad. Sci. U.S.A..

[36] Nagai T, Yamada S, Tominaga T, Ichikawa M, Miyawaki A (2004). Expanded dynamic range of fluorescent indicators for Ca^2+^ by circularly permuted yellow fluorescent proteins. Proc. Natl Acad. Sci. U.S.A..

[37] Nakai J, Ohkura M, Imoto K (2001). A high signal-to-noise Ca(2+) probe composed of a single green fluorescent protein. Nat. Biotechnol..

[38] Yabut (2020). Genetic deletion of mast cell serotonin synthesis prevents the development of obesity and insulin resistance. Nat. Commun..

[39] Diao M, Qu X, Huang S (2018). Calcium imaging in *Arabidopsis* pollen cells using G-CaMP5. J. Int. Plant Biol..

[40] Antoine AF, Faure JE, Dumas C, Feijo JA (2001). Differential contribution of cytoplasmic Ca^2+^ and Ca^2+^ influx to gamete fusion and egg activation in maize. Nat. Cell Biol..

[41] Malho R, Trewavas AJ (1996). Localized apical increases of cytosolic free calcium control pollen tube orientation. Plant Cell.

[42] Boavida LC, Borges F, Becker JD, Feijó J (2011). Whole genome analysis of gene expression reveals coordinated activation of signaling and metabolic pathways during pollen-pistil interactions in Arabidopsis. Plant Physiol..

[43] Vanderstraeten L, Van der Straeten D (2017). Accumulation and transport of 1-aminocyclopropane-1-carboxylic acid (ACC) in plants: current status, considerations for future research and agronomic applications. Front. Plant Sci..

[44] Osborne DJ, Walters J, Milborrow BV, Norville A, Stange LMC (1996). Evidence for a non-ACC ethylene biosynthesis pathway in lower plants. Phytochemistry.

[45] Li F-W (2018). Fern genomes elucidate land plant evolution and cyanobacterial symbioses. Nat. Plants.

[46] VanWent, J. L. & Willemse, M. T. M. *Fertilization*. In *Embryology of Angiosperms* (ed. Johri, B. M.) 273–317 (Springer, 1984).

[47] Linskens HF, Spanjers AW (1973). Changes of the electrical potential in the transmitting tissue of Petunia-styles after cross and self-pollination. Incomp. Newslett..

[48] Spanjers AW (1978). Voltage variation in *Lilium longiflorum* pistils induced by pollination. Experientia.

[49] Spanjers AW (1981). Bioelectric potential changes in the style of *Lilium longiflorum* Thunb. after self- and cross-pollination of the stigma. Planta.

[50] Mousavi SA, Chauvin A, Pascaud F, Kellenberg S, Farmer EE (2013). GLUTAMATE RECEPTOR-LIKE genes mediate leaf-to-leaf wound signaling. Nature.

[51] Toyota M (2018). Glutamate triggers long-distance, calcium-based plant defense signaling. Science.

[52] Leydon AR (2015). Pollen tube discharge completes the process of synergid degeneration that is initiated by pollen tube-synergid interaction in Arabidopsis. Plant Physiol..

[53] Schindelin J (2012). Fiji: an open-source platform for biological-image analysis. Nat. Methods.

[54] Schwab R, Ossowski S, Riester M, Warthmann N, Weigel D (2006). Highly specific gene silencing by artificial microRNAs in Arabidopsis. Plant Cell.

[55] Curtis MD, Grossniklaus U (2003). A gateway cloning vector set for high-throughput functional analysis of genes in planta. Plant Physiol..

[56] Clough SJ, Bent AF (1998). Floral dip: a simplified method for Agrobacterium-mediated transformation of *Arabidopsis thaliana*. Plant J..

[57] Koncz C, Nemeth K, Redei GP, Schell J (1992). T-DNA insertional mutagenesis in *Arabidopsis*. Plant Mol. Biol..

[58] Harrison SJ (2006). A rapid and robust method of identifying transformed *Arabidopsis thaliana* seedlings following floral dip transformation. Plant Methods.

[59] Johnson, M. A. & Kost, B. in *Plant Developmental Biology. Methods in Molecular Biology (Methods and Protocols)*, vol 655 (eds Hennig, L. & Köhler, C.) 155–176 (Humana, 2010).

[60] Mori T, Kuroiwa H, Higashiyama T, Kuroiwa T (2006). Generative cell specific 1 is essential for angiosperm fertilization. Nat. Cell Biol..

[61] Preibisch S, Saalfeld S, Tomancak P (2009). Globally optimal stitching of tiled 3D microscopic image acquisitions. Bioinformatics.

[62] Livak KJ, Schmittgen TD (2001). Analysis of relative gene expression data using real-time quantitative PCR and the 2(-Delta Delta C(T)) method. Methods.

[63] Karimi M, Inzé D, Depicker A (2002). Gateway vectors for Agrobacterium-mediated plant transformation. Trends Plant Sci..

[64] Steffen JG, Kang IH, Macfarlane J, Drews GN (2007). Identification of genes expressed in the Arabidopsis female gametophyte. Plant J..

[65] Wang Z-P (2015). Egg cell-specific promoter-controlled CRISPR/Cas9 efficiently generates homozygous mutants for multiple target genes in Arabidopsis in a single generation. Genome Biol..

[66] Jiang W (2013). Demonstration of CRISPR/Cas9/sgRNA-mediated targeted gene modification in Arabidopsis, tobacco, sorghum and rice. Nucleic Acids Res..

[67] Hellens RP, Edwards EA, Leyland NR, Bean S, Mullineaux PM (2000). pGreen: a versatile and flexible binary Ti vector for Agrobacterium-mediated plant transformation”. Plant Mol. Biol..

[68] Hamill OP, Marty A, Neher E, Sakmann B, Sigworth FJ (1981). Improved patch-clamp techniques for high-resolution current recording from cells and cell-free membrane patches. Pflügers Arch..

[69] Palmer AE, Tsien RY (2006). Measuring calcium signaling using genetically targetable fluorescent indicators. Nat. Protoc..

[70] Shankar S, Calvert MEK, Yew JY (2016). Measuring physiological responses of *Drosophila* sensory neurons to lipid pheromones using live calcium imaging. J. Vis. Exp..

