# Supplementary material for: Ethylene-independent signaling by the ethylene precursor ACC in Arabidopsis ovular pollen tube attraction
Source: Nat Commun. 2020 Aug 14;11:4082. doi: 10.1038/s41467-020-17819-9 (PMC7429864; doi:10.1038/s41467-020-17819-9)
Supplement: Supplementary file 1 — Supplementary Information [file 41467_2020_17819_MOESM1_ESM.pdf]

Supplementary Information

Ethylene-independent signaling by the ethylene precursor ACC in ovular pollen tube attraction in *Arabidopsis*

Mou *et al.*

Supplementary Figure 1

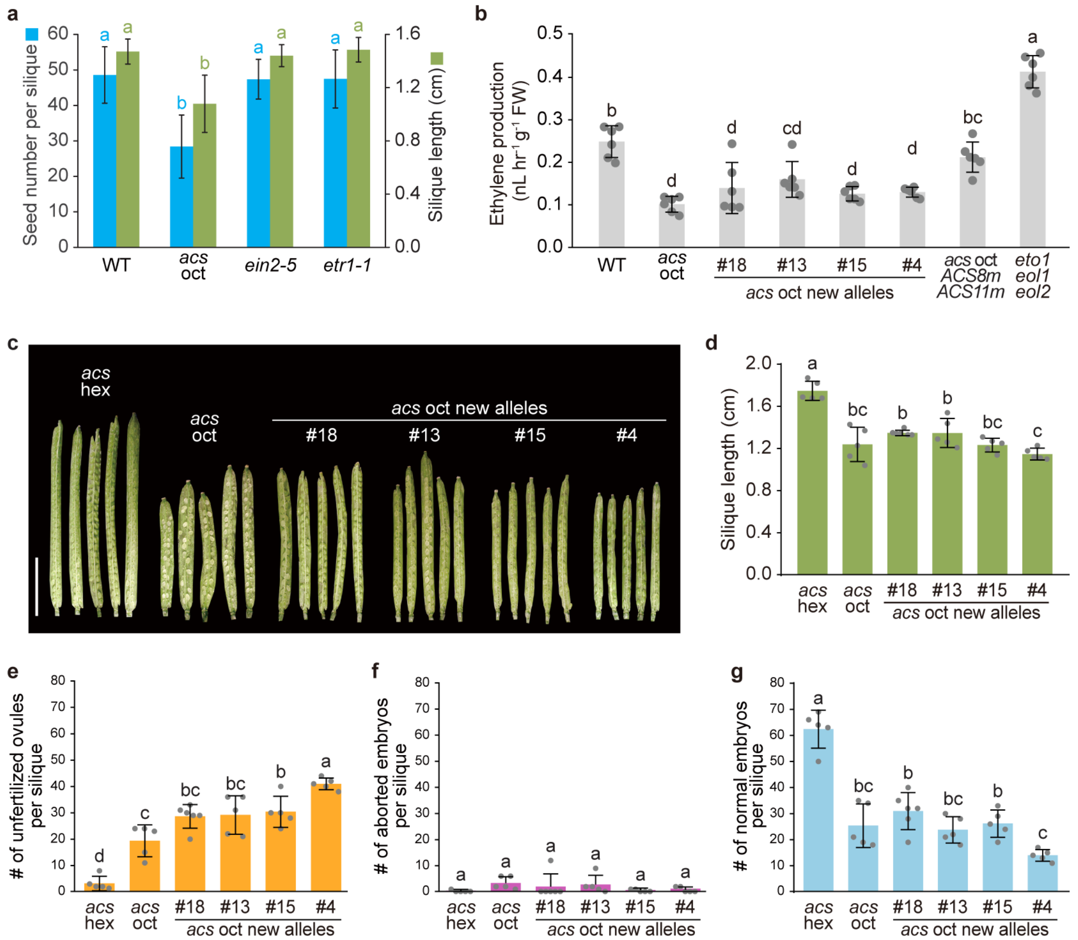

Supplementary Figure 1. *acs* octuple (oct) mutant alleles have fewer seeds (a) and shorter siliques (a, c, d), produce less ethylene (b), and have a higher proportion of unfertilized ovules compared to the wild type (WT) or *acs* hextuple (hex) mutant (e-g). a Average seed number and silique length of *asc* oct[7] compared to WT, *ein2-5*, and *etr1-1*. $n = 30$ siliques per genotype. Different letters indicate significant difference, $P < 0.05$ (one-way ANOVA with Tukey HSD post-hoc test for seed number, df = 3, F value = 47.08; Welch ANOVA with Tamhane's T2 post-hoc test for silique length, df = 3, W = 31.51). **b** Average ethylene production in seedlings. The four new alleles (#18, #3, #15, #4) are compared to the original mutant (*asc* oct). The rescued line *acs* oct carrying transgenes *ACS8m*, *ACS11m* (*acs* oct, *ACS8m*, *ACS11m*) is further analyzed in Supplementary Fig. 2. The positive controls is an ethylene overproducing mutant (*eto1, eol1, eol2*)[1]. $n = 6$ biological replicates per genotype. Different letters indicate significant difference, $P < 0.05$ (one-way ANOVA with Tukey's HSD post-hoc test, df = 5, F = 48.59). **c** Dissected siliques. Scale bar, 5 mm. **d** Average silique length. $n = 5$ siliques per genotype, except for line 18, $n = 6$. Different letters indicate significant difference, $P < 0.05$ (one-way ANOVA with Tukey's HSD post-hoc test, df = 5, F = 22.78). **e-g** Average number of post-pollination ovule fates per silique. $n = 5$ siliques per genotype, except for line 18, $n = 6$. For unfertilized ovules (**e**), different letters indicate significant difference, $P < 0.05$ (one-way ANOVA with Tukey's HSD post-hoc test, df = 5, F = 31.64). For

aborted embryos (**f**), no significant difference was observed (Kruskal-Wallis test, df = 5, Kruskal-Wallis statistic = 11.88). For normal embryos (**g**), different letters indicate significant difference, $P < 0.05$ (one-way ANOVA with Tukey's HSD post-hoc test, df = 5, F = 35.26). Error bars show ± s.d., except in (**f**), error bars show + s.d.

Supplementary Figure 2

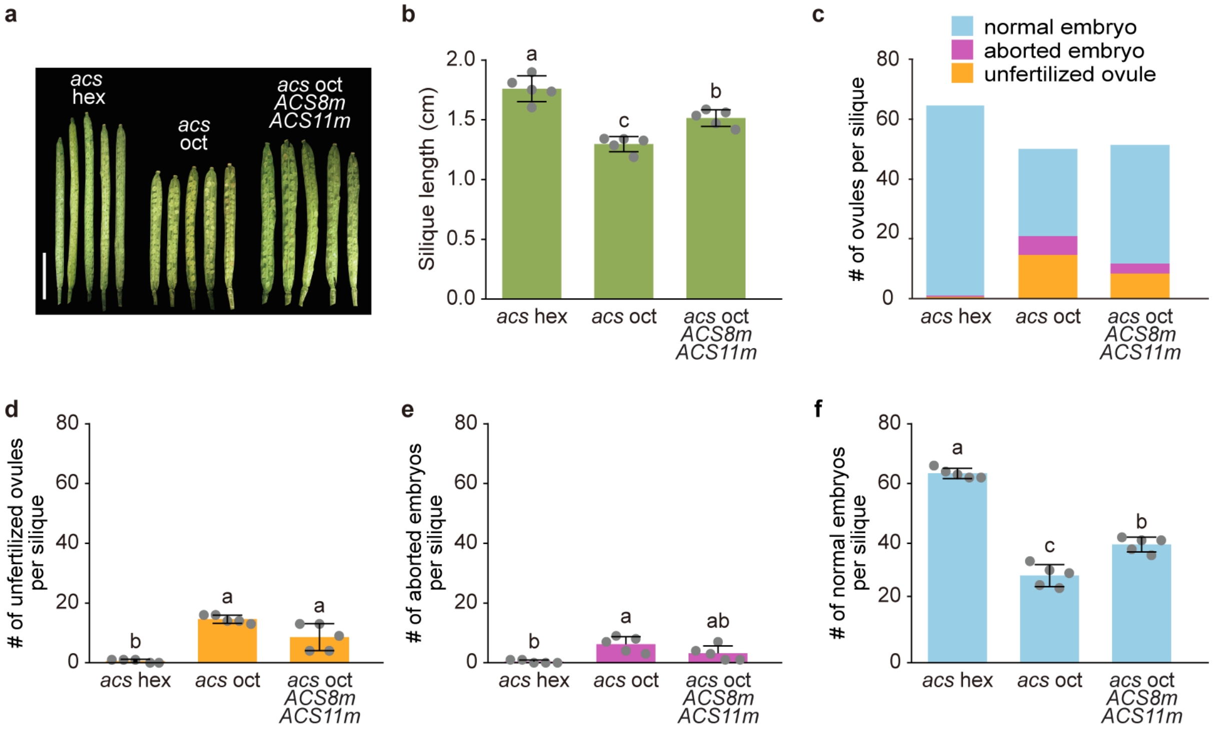

Supplementary Figure 2. Partial rescue of the *acs* oct mutant[7] by co-expression of *ACS8m* and *ACS11m* transgenes. a Dissected siliques. Scale bar, 5 mm. **b** Average silique length. *n* = 5 siliques per genotype. Different letters indicate significant difference, $P < 0.05$ (one-way ANOVA with Tukey's HSD post-hoc test, df = 2, F value = 39.11). **c** Summary of the average number of post-pollination ovule fates per silique in the complemented line (*acs* oct + *ACS8m, ACS11m*) with statistical data shown in **d-f**. *n* = 5 siliques per genotype. For unfertilized ovules (**d**), different letters indicate significant difference, $P < 0.05$ (Welch ANOVA with Tamhane's T2 post-hoc test, df = 2 and W = 214.40). For aborted embryos (**e**), different letters indicate significant difference, $P < 0.05$ (one-way ANOVA with Tukey's HSD post-hoc test, df = 2, F = 9.56). For normal embryos (**f**), different letters indicate significant difference, $P < 0.05$ (one-way ANOVA with Tukey's HSD post-hoc test, df = 2, F value = 202.2). Error bars show ± s.d., except in **e**, error bars show + s.d.

Supplementary Figure 3

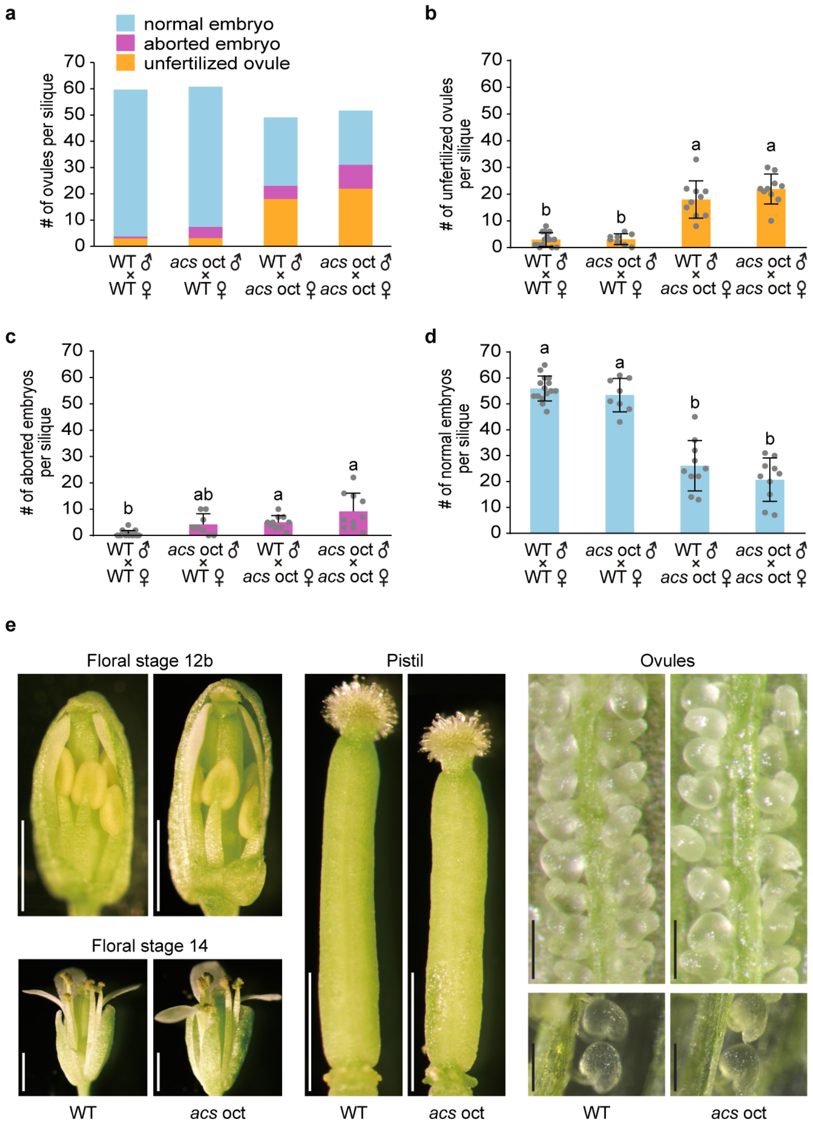

Supplementary Figure 3. Reciprocal crosses indicate a defect in the *acs* oct female (a-d), which shows normal flower morphology (e). a Summary of the average number of post-pollination ovule fates per silique in the F_1 progeny of reciprocal hand-crosses between WT and *acs* oct[7] with statistical data shown in **b-d**. *n* = 15 siliques for WT♀ × WT♂, *n* = 8 for WT♀ × *acs* oct♂, *n* = 10 for *acs* oct♀ × WT♂, *n* = 10 for *acs* oct♀ × *acs* oct♂. For unfertilized ovules (**b**), different letters indicate significant difference, $P < 0.05$ (Kruskal-Wallis test with Dunn's post-hoc test, df =3, Kruskal-Wallis statistic = 32.03). Error bars show ± s.d. For aborted embryos (**c**), different letters indicate significant difference, $P < 0.05$ (Kruskal-Wallis test with Dunn's post-hoc test, df =3, Kruskal-Wallis statistic = 22.01). Error bars show + s.d. For normal embryos (**d**), different letters indicate significant difference, $P < 0.05$ (one-way ANOVA with Tukey's HSD post-hoc test, df = 3, F = 67.56). Error bars show ± s.d. **e** Representative images of *acs* oct mutant flowers compared to

WT flowers with some or all outer organs removed. *Left*: flowers at stage 12b and stage 14 (scale bar, 1mm). *Middle* and *right*: Pistils (scale bar, 1 mm) and ovules (scale bar, 200 µm), 2 days post-emasculation of stage 12b flowers. Pistils of the mutant typically appeared shorter than WT pistils.

Supplementary Figure 4

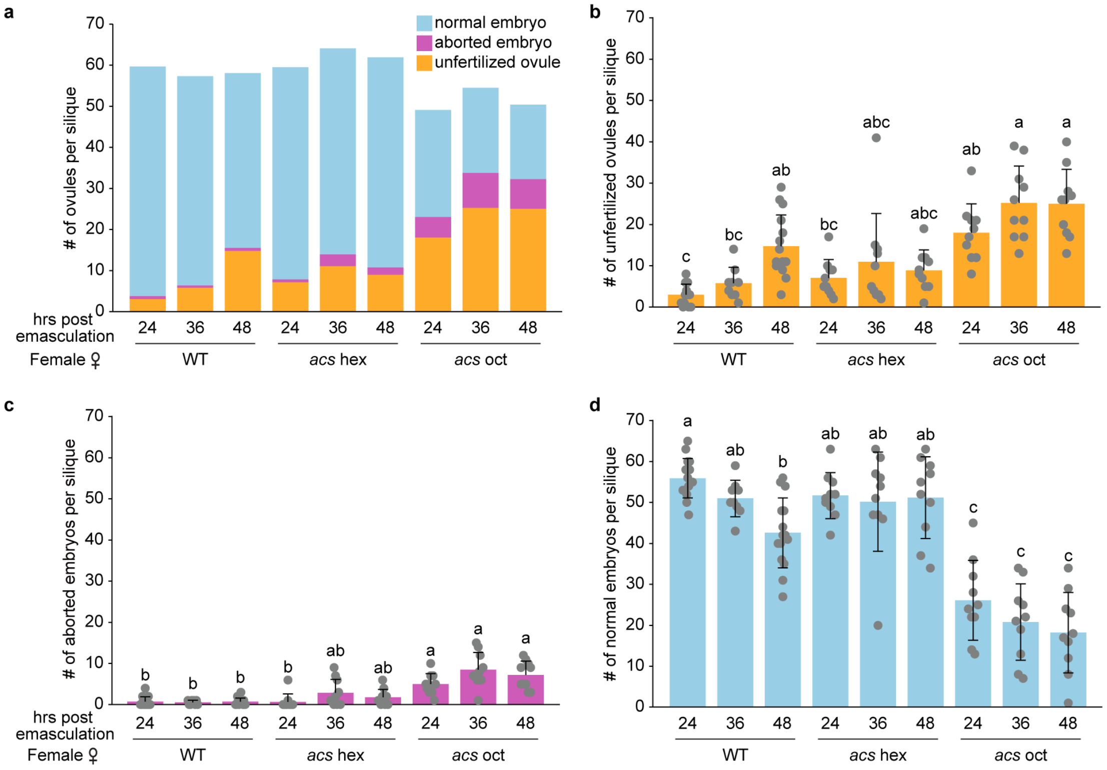

Supplementary Figure 4. Hand-pollination at delayed time points does not increase *acs* oct mutant seed number. a Summary of the average number of post-pollination ovule fates per silique in the F_1 progeny after hand crossing with WT pollen at 24 hrs, 36 hrs, and 48 hrs after emasculation of stage 12b flowers, with statistical data shown in **b-d**. For WT♀×WT♂, *n* = 15 siliques (24 hr), *n* = 9 (36 hr), and *n* = 15 (48 hr). For *acs* hex♀×WT♂ and *acs* oct♀×WT♂, *n* = 10 at all timepoints. For unfertilized ovules (**b**), different letters indicate significant difference, $P < 0.05$ (Kruskal-Wallis test with Dunn's post-hoc test, df = 8, Kruskal-Wallis statistic = 61.84). Error bars show + s.d. For aborted embryos (**c**), different letters indicate significant difference, $P < 0.05$ (Kruskal-Wallis test with Dunn's post-hoc test, df = 8, Kruskal-Wallis statistic = 56.9). Error bars show + s.d. For normal embryos (**d**), different letters indicate significant difference, $P < 0.05$ (one-way ANOVA with Tukey's HSD post-hoc test, df = 8, F value = 32.41). Error bars show ± s.d.

Supplementary Figure 5

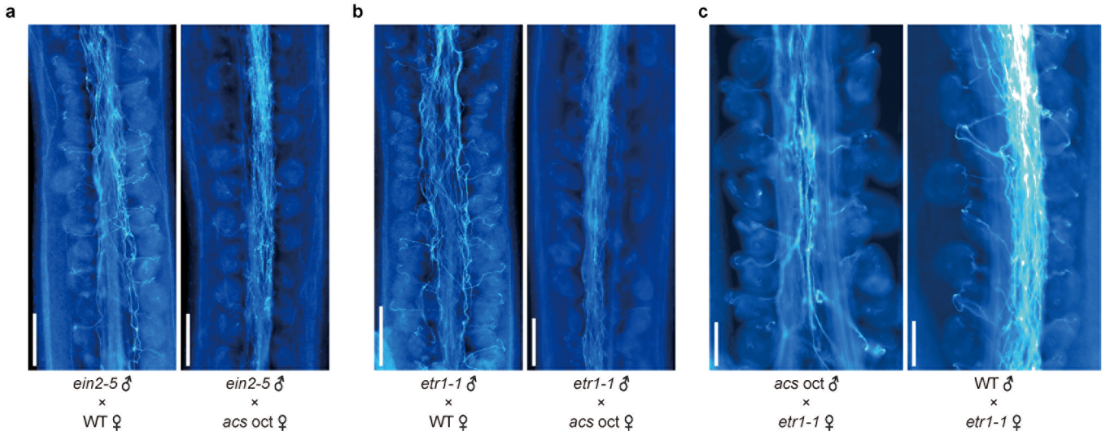

Supplementary Figure 5. Ethylene signaling is unrelated to the pollen tube guidance defect of the *acs* oct female. a and b Representative images of aniline-blue stained pollen tubes of ethylene-insensitive mutants, *ein2-5* (**a**) and *etr1-1* (**b**) in pistils of the WT versus *acs* oct. Scale bar, 200 µm. **c** Representative images of *acs* oct pollen tubes and WT pollen tubes in *etr1-1* pistils. Scale bar, 100 µm.

Supplementary Figure 6

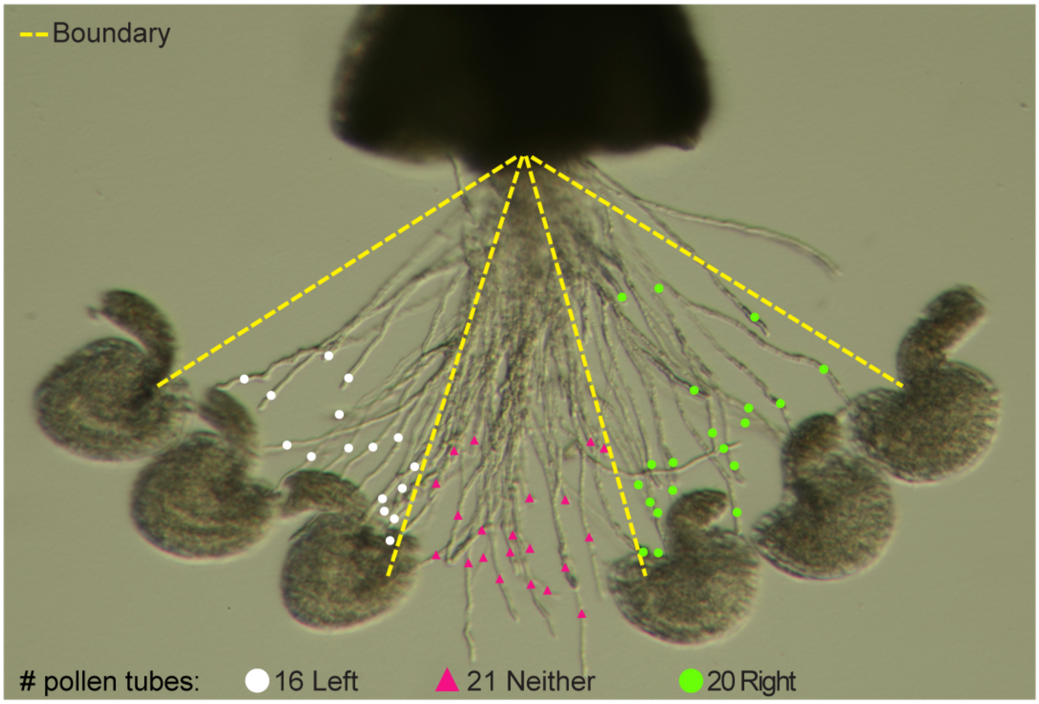

Supplementary Figure 6. Method for scoring pollen tube direction in the semi-*in vivo* assay. The yellow dashed lines indicate the boundaries of the left and right regions extending from the center of the cut style. Pollen tubes whose tips are within the left region are marked with white dots, and those whose tips are within the right region are marked with green dots. Those whose tips show no particular direction (located between the left and right regions) are marked with pink triangles and are counted as "Neither" in Table 1. Pollen tube tips that are out of range (located outside of the two regions on the far left and far right) are not marked and not included in Table 1.

Supplementary Figure 7

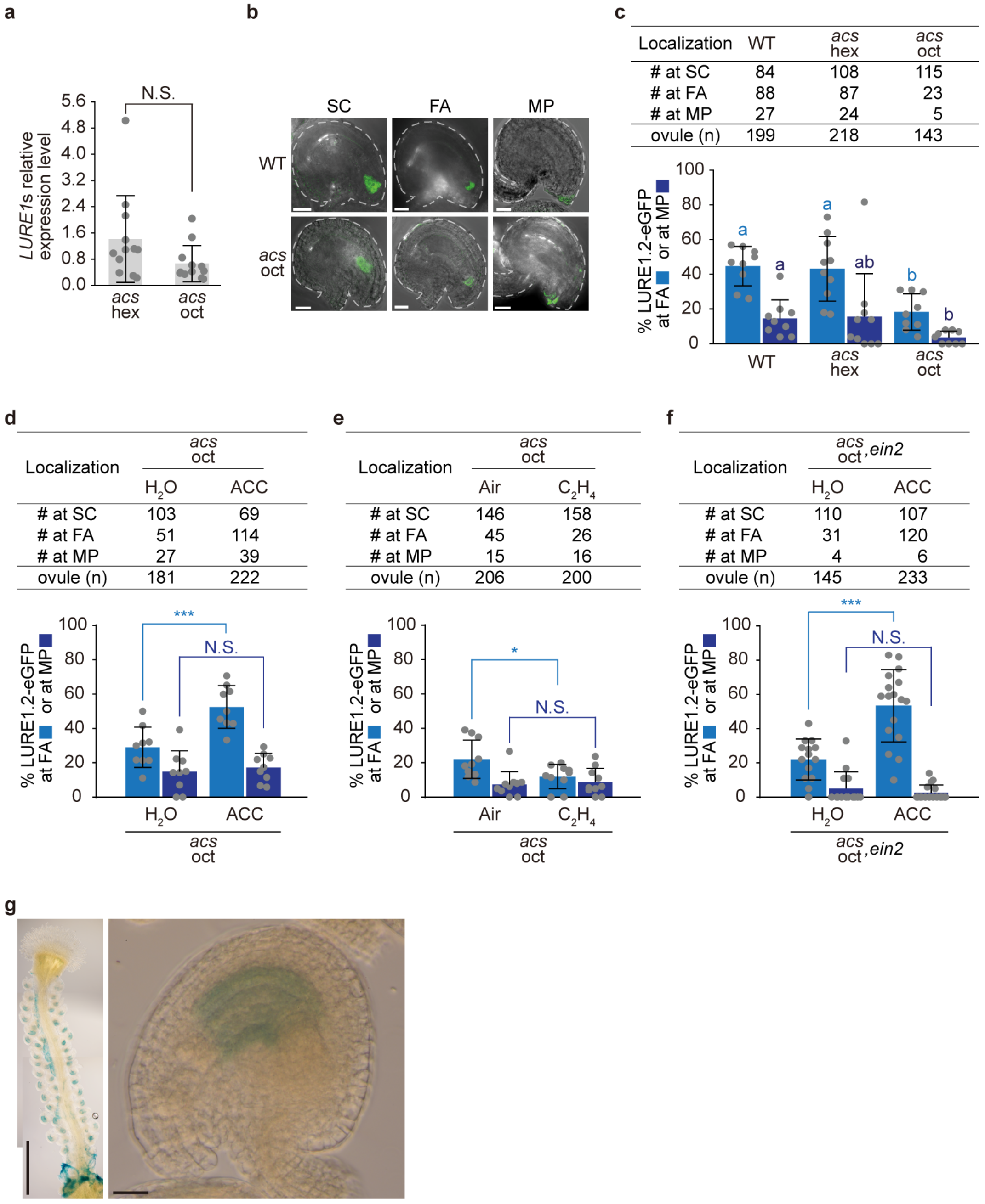

Supplementary Figure 7. LURE1.2-eGFP localization from the SC to the filiform apparatus (FA) is enhanced by ACC and independent of ethylene signaling. a Relative expression (qPCR) of *LURE1*s in *acs* hex and *acs* oct stage 12b flowers shows no significant difference (P = 0.078 by the Mann-Whitney test and P = 0.100 by the Kolmogorov-Smirnov test). n = 12. Error bars show ± s.d. **b** Representative images of LURE1.2-eGFP localization at the SC, FA and micropyle (MP) in WT and *acs* oct mutant ovules 24 hrs post-emasculation of stage 12c flowers. Scale bar = 20 µm. **c-**

f *Top*: numbers of *acs* oct ovules in which LURE1.2-eGFP was observed at the SC, FA, and MP. *Bottom*: average percentages of LURE1.2-eGFP localization in ovules per pistil at the FA (light blue) and MP (dark blue) (for ovules in which localization could be determined). Error bars show ± s.d. for the FA and + s.d. for the MP. **c** No treatment; n = 9, 10, 9 pistils for WT, *acs* hex, *acs* oct, respectively. Different letters indicate significant difference. For the FA comparison, $P < 0.05$ (ANOVA with Tukey's HSD post-hoc test, df = 2, F value = 9.949). For the MP comparison, $P < 0.05$ (Kruskal-Wallis test, df = 2, Kruskal-Wallis statistic = 6.232). **d** Pre-treatment with ACC (1 μM); n = 9 pistils per sample. For the FA comparison, P = 0.0008 (***) (two-tailed t-test, t = 4.123, df=16). For the MP comparison, no significant difference was observed, P = 0.6214 (N.S) (two-tailed t-test, t = 0.504, df=16.). **e** Pre-treatment with C_2H_4 (10 ppm); n = 10 pistils per sample. For the FA comparison, P = 0.0264 (*) (two-tailed t-test, t = 2.418, df=18). For the MP comparison, no significant difference was observed (P = 0.838 by the Mann-Whitney test and P = 0.795 by the Kolmogorov-Smirnov test). **f** Pre-treatment of *acs* oct, *ein2* with ACC (1 μM); n = 14 pistils for H_2O, n = 17 pistils for ACC. For the FA comparison, $P < 0.0001$ (****) (two-tailed t-test with Welch's correction, t value = 5.197, df=26.01.). For the MP comparison, no significant difference was observed (P = 0.722 by the two-tailed Mann-Whitney test and P = 0.824 by the two-tailed Kolmogorov-Smirnov test). **g** Representative GUS staining localization of *proACS8*:GUS in ovules. For the entire dissected pistil (left), scale bar = 500 μm. For the single ovule (right), scale bar = 20 μm.

Supplementary Figure 8

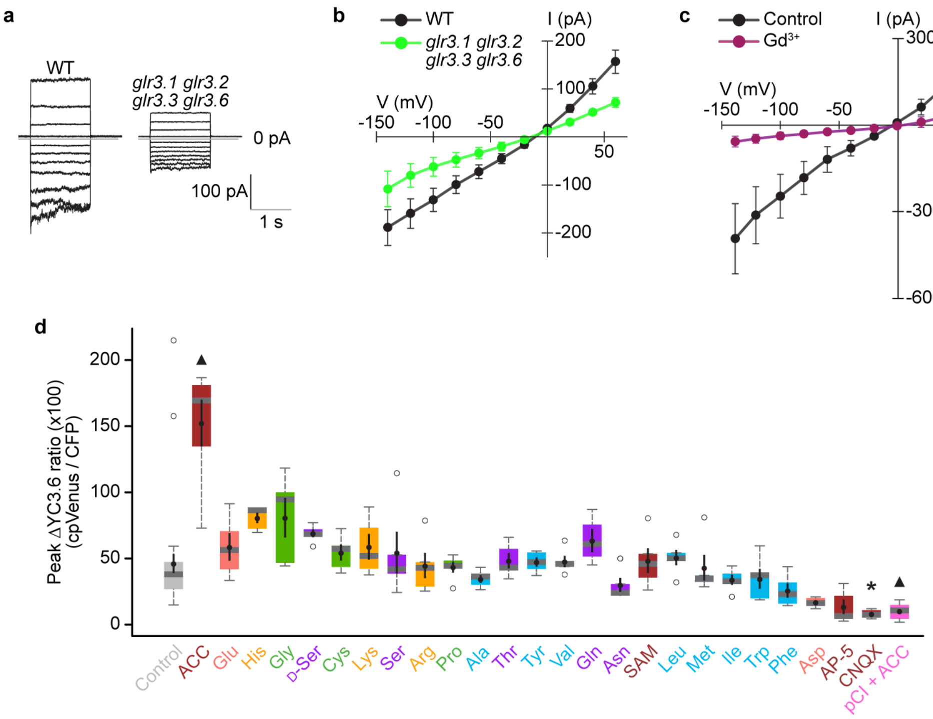

Supplementary Figure 8. Supplementary electrophysiology data indicating stimulation of GLR channels by ACC. a Typical whole cell currents recorded in *Arabidopsis* root protoplasts of the WT (*left*) and *glr3.1, glr3.2, glr3.3, glr3.6* mutant (*right*). 1.6 sec-long pulses were imposed on the protoplasts from -140 mV to +60 mV (20 mV increments), as described in the Methods and used in Figs. 3a, b. **b** Stationary current/voltage curves (I/V) ± s.e. measured in experiments as shown in (**a**). *n* = 18 for wild type, *n* = 7 for the *glr3.1, glr3.2, glr3.3, glr3.6* mutant. **c** Average stationary current/voltage curves (*I/V*) ± s.e. measured by patch clamp under the whole cell configuration in WT root protoplasts in standard solution (black circles) and after addition of 500 µM $GdCl_3$ (purple circles). *n*=4 for each treatment. **d** Comparison of ACC to the 20 proteinogenic amino acids based on Ca^{2+} imaging in COS-7 cells expressing *Pp*GLR1 and YC3.6 as in Figs. 3c and 3d. Changes in cytosolic Ca^{2+} were evaluated by measuring the difference in the peak YC3.6 ratio for each amino acid and presented as box plots (top of the box is 75th percentile; bottom of the box is 25th percentile; horizontal grey bar is median; grey whiskers show the maximum and minimum value within 1.5x the interquartile range) with outliers (open circles) and mean (solid black circles) ± s.e. Included are two candidate ligands, D-Serine (D-Ser) and the ACC precursor S-adenosyl-L-methionine (SAM), and two classic iGluR antagonists, 2-amino-5-phosphonovalerate (AP-5) and 6-cyano-7-nitroquinoxaline-2,3-dione disodium salt hydrate (CNQX). *n* = 31 for pCI-*Pp*GLR1 Control (no amino acid added), *n* = 6 for pCI-*Pp*GLR1+ACC, *n* = 10 for pCI (empty vector) + ACC, *n* = 5 for all other treatments. Statistical significance was determined using Dunnett's test (**P* < 0.05, solid black triangle *P* < 0.001).

Supplementary Figure 9

Localization	*acs* oct H_2O	*acs* oct A23187
# at SC	99	46
# at FA	37	88
# at MP	1	10
ovule (n)	137	144

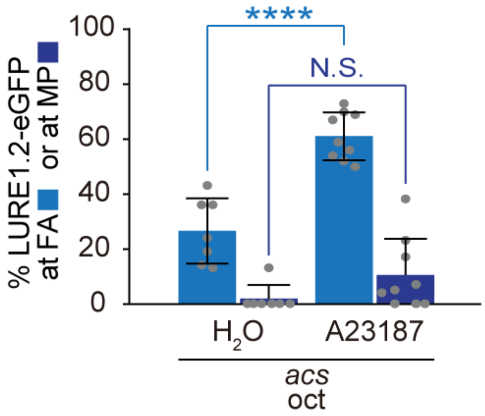

Supplementary Figure 9. Treatment with the calcium ionophore A23187 rescues LURE1.2-eGFP localization in *acs* oct ovules. *Top*: numbers of *acs* oct ovules in which LURE1.2-eGFP was observed at the SC, FA, and MP for the water control and treatment with A23187 (10 µM). *Bottom*: average percentages of LURE1.2-eGFP localization in ovules per pistil at the FA (light blue) and MP (dark blue) (for ovules in which localization could be determined). $n = 7$ pistils for *acs* oct pre-treated with water, $n = 9$ for *acs* oct pre-treated with 10 µM A23187. For the FA comparison, $P < 0.0001$ (****) (two-tailed t-test, t value = 6.742, df=14). For the MP comparison, no significant difference was observed ($P = 0.066$ by the Mann-Whitney test and $P = 0.100$ by the Kolmogorov-Smirnov test). Error bars show ± s.d. for the FA and + s.d. for the MP.

Supplementary Table 1. List of PCR primers used

**Genotyping the gentamycin resistance gene (on the *amiR* transgene)**	
GmR-F	TTGGGTCGATATCAAAGTGCA
GmR-R	TACGCAGCAGCAACGATGTTA
**Genotyping the hygromycin resistance gene**	
Hyg-genotype-F	GGCGACCTCGTATTGGGAAT
Hyg-genotype-R	GAGGGCGTGGATATGTCCTG
**Constructing a new *amiRNA* that targets *ACS8* and *ACS11***	
Generating a new amiRNA using the template pRS300	
Primer A	TCTAGAGGATCCCCGGGTACCTGCAAGGCGATTAAGTTGGGTAAC
Primer-IV	GAAAGGCCTTGATCTTTGGATAATCTACATATATATTCCT
Primer-II	GATTGTCCAAAGATCTAGGCCTATCAAAGAGAATCAATGA
Primer-III	GATTATCCAAAGATCAAGGCCTTTCACAGGTCGTGATATG
Primer-I	GATAGGCCTAGATCTTTGGACAATCTCTCTTTTGTATTCC<br>(The *amiRNA* sequence is underlined)
Primer B	GGTGGGCGGCCGCTCTAGAAGCGGATAACAATTTCACACAGGAAACAG
Genotyping the new amiRNA	
amiRNA-F	AAGACCGGCAACAGGATTCA
amiRNA-R	GATTATCCAAAGATCAAGGCCTTTCACAGGTCGTGATATG
**Generating *ACS8m* and *ACS11m* transgenes for complementing the *acs* octuple mutant**	
Amplifying ACS8m from the promoter region to the stop codon in two overlapping fragments	
*At*ACS8m-F1	GTCGACTGGATCCGGTACCGGCTTAAAGGTCGCAAAGCGCA
*At*ACS8m-R1	**C**GT**T**TC**A**TT**C**GC**T**GGAGTAGCACCAGCGGTGAGGACAAGC<br>(The *amiRNA* sequence is underlined; mutations are in boldface)
*At*ACS8m-F2	CTACTCC**A**GC**G**AA**T**GA**A**AC**G**CTCATGTTTTGTCTCGCTGA<br>(The *amiRNA* sequence is underlined; mutations are in boldface)
*At*ACS8m-R2	ATATCTCGAGTGCGGCCGCGCTATCGTTCCTCGGGTTCAC
Amplifying ACS11m from the promoter region to the stop codon in two overlapping fragments	
*At*ACS11-F1	GTCGACTGGATCCGGTACCGACCGTAAAAAGACATAAACGCT
*At*ACS11-R1	**AC**GT**T**TC**A**TT**G**GC**T**GAGGTTGATCCAGCTGTAAGAACCATC<br>(The *amiRNA* sequence is underlined; mutations are in boldface)
*At*ACS11-F2	CAACCTC**A**GC**C**AA**T**GA**A**AC**GT**TAATGTTCTGTCTTGCTAATCC<br>(The *amiRNA* sequence is underlined; mutations are in boldface)
*At*ACS11-R2	ATATCTCGAGTGCGGCCGCGTCAACGTTCTGATTCACAAG
Amplifying the OCS terminator to clone into pMD99	
OCS-terminator-F	TTCGATAATTCCTTAATTAACTGCTTTAATGAGATATGCGAGAC
OCS-terminator-R	CATGATTACGAATTCGAGCTCTGCTGAGCCTCGACATGTT
Genotyping the proACS8:ASC8m and *proACS11:ASC11m transgene (generates a 2324 bp fragment)*	
*At*ACS8seq-2R	AGTGTCCCATTGTAAGCACG
*At*ACS11seq-2R	GGGTCATAGCCTCATAGGTA
**qPCR of *LURE1s***	
LURE1s-qPCR-F	TTGTTTCTTCATGTACATCA
LURE1s-qPCR-R	ATACAAGCTCTTGCGCAATC
**Assembling the *proLURE1.2:LURE1.2-eGFP* plasmid**	
Amplifying the original plasmid backbone sequence	
TP45_pEG-F1	ACAGAGGCAAGAGCAGCAGCTGACGCGTACACAACAAGTCAGCAAACAGACAGGTTG
EP09_at-DD2-eGFP-R1	ATAAGAAACAGAGATAGCCCTTTGGTCTTCTGAGACTG

Amplifying the LURE1.2 promoter and coding sequence	
TP101	TCAGAAGACCAAAGGGCTATCTCTGTTTCTTATCAGAATACTTTTACTTGTC
TP102	TCGCCCTTGCTCACCATTTTAATATCACTAATACTGCAACGAC
Amplifying the eGFP coding sequence	
TP103	TAGTGATATTAAAATGGTGAGCAAGGGCGAGGAGCTGTTC
TP104	AGTCCCGGGTCTTAATTAACTCTCTAGACTTACTTGTACAGCTCGTCCATGCC
Amplifying the coding sequence for hygromycin resistance	
TP114_HygR-F	ACCAATCTCGATACACCAAATCGAATCGATGAAAAAGCCTGAACTCACCGCG
TP113_HygR-R	AACATGGGAGTCCAAGATTCTGCAGCTATTTCTTTGCCCTCGGACGAG
**Knocking out *EIN2* by CRISPR/Cas9 in the *acs* octuple mutant**	
Amplifying the Gateway cassette from a modified pHEE401E[2] template	
forward	CACTATAGGGCGAATTGGGTACCGACGCACTAGTGATATCACAAG
reverse	CAAACGCAAATGCTTTTATTCACTAGTCAAGCTTTCACCACTTTGTAC
EIN2 guide RNAs	
gRNA1	GTGAATGTGAGACCTCAGCT
gRNA2	GTGCATGTTCTTGGGCATTC
Amplifying four fragments from pCAMBIA1302-Cas9-sgRNA[3]	
01-(F)	TGGCGCCGGAACCAATTCAGGGGAGCTCGAATTCCCAT
02-(R)	AGCTGAGGTCTCACATTCACAATCACTACTTCGACTCTAGCTGT
03-(F)	GTGAATGTGAGACCTCAGCTGTTTTAGAGCTAGAAATAGCAAGT
04-(R)	TCTAGAAGGCCTGGATCCATCGATAAAAATTATATCCTGTGGTCGT
05-(F)	ATCGATGGATCCAGGCCTTCTAGAGTAAAGCCTGTAGAAGAGGTTTC
06-(R)	GAATGCCCAAGAACATGCACAATCACTACTTCGACTCTAGCTGT
07-(F)	GTGCATGTTCTTGGGCATTCGTTTTAGAGCTAGAAATAGCAAGT
08-(R)	AGCTGGGTCTAGATATCTCGAGTCAAACACTGATAGTTTAATTGG
Identifying deletions in EIN2	
CRISPR-ein2-F	ACCACGACTGTAGAGTTGACCTTG
CRISPR-ein2-R	GGTTAAGTGCATGCGCAACT

Supplementary References

1. Christians, M. J. *et al*. The BTB ubiquitin ligases ETO1, EOL1 and EOL2 act collectively to regulate ethylene biosynthesis in Arabidopsis by controlling type-2 ACC synthase levels. *Plant J.* **57**, 332-345 (2009).
2. Wudick, M. M. *et al*. CORNICHON sorting and regulation of GLR channels underlie pollen tube Ca^{2+} homeostasis. *Science* ***360,*** 533-536 (2018).
3. Jiang, W. *et al.* Demonstration of CRISPR/Cas9/sgRNA-mediated targeted gene modification in Arabidopsis, tobacco, sorghum and rice, *Nucleic Acids Res.* **41**, e188 (2013).
