# Supplementary material for: Ethylene-independent signaling by the ethylene precursor ACC in Arabidopsis ovular pollen tube attraction
Source: Nat Commun. 2020 Aug 14;11:4082. doi: 10.1038/s41467-020-17819-9 (PMC7429864; doi:10.1038/s41467-020-17819-9)
Supplement: Supplementary file 2 — Descriptions of Additional Supplementary Files [file 41467_2020_17819_MOESM2_ESM.docx]

**Descriptions of Additional Supplementary Files**

**Supplementary Movie 1.**

**Description:** Ca^2+^ oscillations in *ein2-5* ovules upon treatment with 500 μM ACC. Time series of Ca^2+^ dynamics visualized by GCaMP3 corresponding to the top row of images in Fig. 4a. Images were captured at 10-sec intervals, and the video is displayed at 7 frames per second.
 
**Supplementary Movie 2.**

**Description:** Ca^2+^ oscillations in the *ein2-5*ovules upon treatment with 500 μM ACC applied after 10 min treatment with 50 μM CNQX. Time series of Ca^2+^ dynamics visualized by GCaMP3 corresponding to the bottom row of images in Fig. 4a. Images were captured at 10-sec intervals, and the video is displayed at 7 frames per second.
