# Supplementary material for: Ethylene-independent signaling by the ethylene precursor ACC in Arabidopsis ovular pollen tube attraction
Source: Nat Commun. 2020 Aug 14;11:4082. doi: 10.1038/s41467-020-17819-9 (PMC7429864; doi:10.1038/s41467-020-17819-9)
Supplement: Supplementary file 5 — Reporting Summary [file 41467_2020_17819_MOESM5_ESM.pdf]

nature research

Corresponding author(s): Caren Chang, José Feijó

Last updated by author(s): Jul 27, 2020

Reporting Summary

Nature Research wishes to improve the reproducibility of the work that we publish. This form provides structure for consistency and transparency in reporting. For further information on Nature Research policies, see Authors & Referees and the Editorial Policy Checklist .

Statistics

For all statistical analyses, confirm that the following items are present in the figure legend, table legend, main text, or Methods section.

n/a	Confirmed	
☐	☒	The exact sample size (*n*) for each experimental group/condition, given as a discrete number and unit of measurement
☐	☒	A statement on whether measurements were taken from distinct samples or whether the same sample was measured repeatedly
☐	☒	The statistical test(s) used AND whether they are one- or two-sided<br>*Only common tests should be described solely by name; describe more complex techniques in the Methods section.*
☒	☐	A description of all covariates tested
☐	☒	A description of any assumptions or corrections, such as tests of normality and adjustment for multiple comparisons
☐	☒	A full description of the statistical parameters including central tendency (e.g. means) or other basic estimates (e.g. regression coefficient) AND variation (e.g. standard deviation) or associated estimates of uncertainty (e.g. confidence intervals)
☐	☒	For null hypothesis testing, the test statistic (e.g. *F*, *t*, *r*) with confidence intervals, effect sizes, degrees of freedom and *P* value noted<br>*Give P values as exact values whenever suitable.*
☒	☐	For Bayesian analysis, information on the choice of priors and Markov chain Monte Carlo settings
☒	☐	For hierarchical and complex designs, identification of the appropriate level for tests and full reporting of outcomes
☒	☐	Estimates of effect sizes (e.g. Cohen's *d*, Pearson's *r*), indicating how they were calculated

Our web collection on statistics for biologists contains articles on many of the points above.

Software and code

Policy information about availability of computer code

Data collection	Patch-clamp data were collected using Axon pClampex6 from the pClamp6 software suite (Molecular Devices), and Ca^{2+} imaging in ovules was captured using Micro-Manager 1.4.16.
Data analysis	ImageJ was used to process Ca^{2+} imaging data, measure silique length, and create composite images of existing pollen tube staining images. Axon Clampfit6 from the pClamp6 software suite (Molecular Devices) was used for patch-clamp data analyses.

For manuscripts utilizing custom algorithms or software that are central to the research but not yet described in published literature, software must be made available to editors/reviewers. We strongly encourage code deposition in a community repository (e.g. GitHub). See the Nature Research guidelines for submitting code & software for further information.

Data

Policy information about availability of data

All manuscripts must include a data availability statement. This statement should provide the following information, where applicable:

- Accession codes, unique identifiers, or web links for publicly available datasets
- A list of figures that have associated raw data
- A description of any restrictions on data availability

Source data are provided with this paper as a Source Data file. Other data and biological materials are available from the corresponding authors upon reasonable request.

Field-specific reporting

Please select the one below that is the best fit for your research. If you are not sure, read the appropriate sections before making your selection.

Life sciences study design

All studies must disclose on these points even when the disclosure is negative.

Sample size	Sample sizes are provided in the figure legends and were based on common standards in the field, as in the patch-clamp and Ca^{2+} imaging studies.
Data exclusions	For Ca^{2+} imaging in ovules and in COS-7 cells, non-healthy-looking ovules and cells, respectively, were excluded from the data analysis. In imaging of LURE1.2-GFP in ovules, ovules in which GFP localization could not be confidently determined were excluded from the data analysis.
Replication	All experiments were replicated, and all replication attempts were successful.
Randomization	For measurements/assays using plants or root protoplasts, allocation to experimental groups was based on genotype.
Blinding	For the assessment of LURE1.2-eGFP localization, we used blind tests for +/- ACC treatment. Additionally, independent assessors scored LURE1.2-eGFP localization in the same samples.

Reporting for specific materials, systems and methods

We require information from authors about some types of materials, experimental systems and methods used in many studies. Here, indicate whether each material, system or method listed is relevant to your study. If you are not sure if a list item applies to your research, read the appropriate section before selecting a response.

Materials & experimental systems

n/a	Involved in the study	
☒	☐	Antibodies
☐	☒	Eukaryotic cell lines
☒	☐	Palaeontology
☒	☐	Animals and other organisms
☒	☐	Human research participants
☒	☐	Clinical data

Methods

n/a	Involved in the study	
☒	☐	ChIP-seq
☒	☐	Flow cytometry
☒	☐	MRI-based neuroimaging

Eukaryotic cell lines

Policy information about cell lines

Cell line source(s)	Cells were purchased from two commercial sources (ATCC and Sigma-Aldrich)
Authentication	None of the cell lines were authenticated
Mycoplasma contamination	Cell lines were not tested for mycoplasma contamination
Commonly misidentified lines (See ICLAC register)	*Name any commonly misidentified cell lines used in the study and provide a rationale for their use.*
